# *Heterorhabditis caligo* n. sp. (Rhabditida: Heterorhabditidae): A New Entomopathogenic Nematode from Pichilemu Sand Dunes, Chile

**DOI:** 10.2478/jofnem-2025-0045

**Published:** 2025-11-10

**Authors:** Ernesto San-Blas, Patricia Morales-Montero, Brynelly Bastidas, Vladimir Půža, Ricardo A. R. Machado

**Affiliations:** Laboratory of Nematology, Institute of Agri-food, Animal and Environmental Sciences (ICA3), Universidad de O′Higgins, Campus Colchagua,Chile; Centre of Systemic Biology for Crop Protection (Biosav-UOH), Universidad de O’Higgins, Campus Colchagua,Chile; Institute of Entomology, Biology Centre of the Czech Academy of Sciences, CAS, České Budějovice 37005, Czech Republic; Faculty of Agriculture and Technology, University of South Bohemia, České Budějovice 37005, Czech Republic; Computational Biology, Independent Researcher, Medellin, Colombia

**Keywords:** description, molecular biology, morphology, morphometry, phylogeny, symbiotic bacteria

## Abstract

During a survey of the nematode biodiversity in the Petrel wetland (central Chile), a population of *Heterorhabditis* sp. was found in the coastal dune samples. Morphological, morphometric, and molecular studies indicated that this nematode belonged to the *megidis* group, and represented a novel species, which we named *Heterorhabditis caligo* n. sp. This nematode species resembles *H. marelatus* but it is different in the morphometrics of its infective juvenile in the following ways: pharynx length (135–150 μm vs. 120–138 μm), and the position of the excretory pore from the anterior end (105–128 μm vs. 81–113 μm). In males, the fourth and eighth pairs of the bursal papillae are shorter and do not reach the edge of the bursa in *H. caligo* n. sp., whereas all the papillae in *H. marelatus* reach the edge of the bursa. The excretory pore of amphimictic females of *H. caligo* n. sp. is located more posteriorly than in those of *H. marelatus* 193 (169–224) μm vs. 157 (139–178) μm, respectively. Phylogenetic analyses of the genus based on whole nuclear and mitochondrial genome sequences and on five gene markers showed a clear separation of *Heterorhabditis caligo* n. sp. from the other species, placing it within the *megidis* group.

Entomopathogenic nematodes (EPNs) from the genus *Heterorhabditis* Poinar (1927) have been used as biological control agents for many insect pests around the globe due to their ease of mass production, high virulence, and host specificity. Their effective performance in the field has led to increased research of these organisms, especially in many of the developing countries (San-Blas et al, 2019; van Lenteren et al., 2025).

*Heterorhabditis* have been found in many regions and habitats from all over the world, except in Antarctica (Griffin et al., 1990). Currently, 22 *Heterorhabditis* species have been described (Půža and Machado, 2024; Machado et al., 2025a, 2025b; Půža et al., 2025) and, according to phylogenetic analysis, the genus is divided into three groups: *indica*, *bacteriophora*, and *megidis* (Nguyen et al., 2008; Dhakal et al., 2020; Půža and Machado, 2024). Despite surveys in 7 out of 13 South American countries, the prevalence of EPNs and their biodiversity remain largely unknown across the subcontinent, and published information is limited (San-Blas et al., 2019). In South America, five *Heterorhabditis* species have been reported or described: *Heterorhabditis hambletoni* (Pereira, 1937), *H. amazonensis* (Andaló et al., 2006), *H. indica* (Poinar et al., 1992), *H. atacamensis* (Edgington et al., 2011), and *H. bacteriophora* (Poinar, 1975), while the latter two species have been found in Chile (Edgington et al., 2011; San-Blas et al., 2019; Lankin et al., 2022).

During a survey on the biodiversity of nematodes in the Petrel lagoon in 2023 (Pichilemu, central Chile), a population of *Heterorhabditis* sp. was found in a coastal dune (between the shore and the lagoon). Morphological, morphometric, and molecular analyses indicated that the recovered nematodes represent a new species that belongs to the *megidis* group. We describe this new species herein as *Heterorhabditis caligo* n. sp.

## Materials and Methods

### Sampling procedure

*Heterorhabditis caligo* n. sp., was isolated from a coastal dune between the Petrel lagoon and the seashore in Pichilemu (central Chile) in January 2023. A composite soil sample (20 subsamples of 1 kg each) was baited using *Galleria mellonella* (L.) (Lepidoptera: Pyralidae) larvae (Bedding and Akhurst, 1975). The soil samples with the *G. mellonella* larvae were kept in the dark at 20°C; after 7 days, dead larvae showing red coloration (typical for *Heterorhabditis* infection) were recovered and placed in White traps (White, 1927). Emerging infective juveniles (IJs) from the traps were stored at 15°C and a sample was used to reinfect fresh *G. mellonella* to confirm Koch’s postulates.

### Morphologic characterization

For taxonomic studies, 10 *G. mellonella* larvae were exposed to IJs (100 IJs per *G. mellonella*) in a 9.0 cm diameter Petri dish lined with moistened filter paper and kept in the dark at 20°C. Hermaphrodites were collected 5 days post-infection; males and amphimictic females were collected 7 days post-infection by dissecting the *G. mellonella* cadavers in tap water, IJs were obtained from *G. mellonella* cadavers placed on White traps (White, 1927), and nematodes were collected within the first week of emergence.

Live observations of hermaphrodites, males, females, and IJs were carried out using light microscopy. For more detailed studies and the preparation of permanent slides, 20 additional specimens per stage were collected in tap water and killed by heating at 60°C for 5 min and immediately fixed with TAF (7 ml formalin, 2 ml triethanolamine, 91 ml distilled water) at the same temperature (Courtney et al., 1955). Fixed nematodes were processed for glycerine mounting, using a slow evaporation technique. Permanent slides were prepared using glass slides and cover-glass supported with Parafilm strips to prevent flattening. The slides were sealed with nail polish. These mounted nematodes were used for morphometric studies and description, while morphometrics of IJs were done using live and dead specimens mounted in tap water (Nguyen, 2007) using a Leica DM2500 compound microscope equipped with differential interference contrast.

## Molecular Characterization

### Nematode nuclear and mitochondrial genome sequencing and assembly procedures

Genome sequencing was carried out largely based on the work of Machado et al. (2025a, 2025b), with minor modifications. Briefly, genomic DNA of *H. caligo* n. sp. UOH-032 was extracted using a Blood and Tissue Kit (Qiagen, Hilden, Germany). The resulting DNA was used for library preparation using the VAHTS^®^ Universal Plus DNA Library Prep Kit for Illumina (Ref. ND617, Vazyme Biotech Co., Nanjing, China). Indexed libraries were then pooled at equimolar concentrations and sequenced (2 × 150 bp) on an Illumina NovaSeq 6000 instrument. High-quality reads were obtained using fastp v0.23.4 (Chen et al., 2018). The resulting reads were assembled with SPAdes 3.15.5 (Bankevich et al., 2012). Scaffolds shorter than 200 bp were removed. The final assemblies were polished using Pilon 1.24 and the National Center for Biotechnology Information (NCBI) Foreign Contamination Screen (FCS) genome cross-species aligner (GX) tool (NCBI FCS GX v0.5.0) was used to remove scaffolds belonging to organisms other than Nematoda (Walker et al., 2014; Astashyn et al., 2024). Finally, nuclear genome completeness and contamination were assessed using BUSCO 5.4.6 and the nematoda_odb10 database (Seppey et al., 2019). The mitochondrial genome of *H. caligo* n. sp. UOH-032 was assembled using NOVOplasty v4.3.1 (Dierckxsens et al., 2016). Features of the assembled genome of *H. caligo* n. sp. UOH-032 and the genomes of other *Heterorhabditis* species used in this study and related statistics are presented in Tables S1 and S2 in the Supplementary Material.

### Nematode whole nuclear and mitochondrial phylogenomic reconstructions

Phylogenomic and phylogenetic reconstructions were carried out largely based on the work of Machado et al. (2025a, 2025b) with minor modifications. To estimate the evolutionary relationships based on nuclear genomes, ortholog clustering analyses were first performed using OrthoFinder2 on the protein-coding genes of all the *Heterorhabditis* species, with *Oscheius tipulae* as the outgroup (Emms and Kelly, 2019). Protein sequences of *H. caligo* n. sp. UOH-032 were obtained by annotating the assembled genomes using Maker v2.31.11 (Cantarel et al., 2008). After the ortholog clustering analyses, single-copy orthogroups were selected and their sequences were aligned with MAFFT v7.526 (Katoh et al., 2005). The resulting alignment was then used to reconstruct phylogenetic relationships using FastTree v2.1.10 based on the Jones-Taylor-Thornton 1992 model with a continuous approximation of the gamma distribution (JTT + CAT) (Price et al., 2010). To estimate the evolutionary relationships based on mitochondrial genomes, the mitochondrial genomes were first assembled using NOVOplasty v4.3.1 and then annotated using MITOS2 v2.1.9 (Bernt et al., 2013; Dierckxsens et al., 2016). Protein-coding genes were then concatenated in the following order: cytochrome c oxidase subunits 1–3 (*cox-1*, *cox-2*, and *cox-3*), cytochrome b (*cob*), and NADH dehydrogenase subunits 1–6 (*nad-1*, *nad-2*, *nad-3*, *nad-4*, *nad-4l*, *nad-5*, and *nad-6*). The obtained concatenated sequences were then aligned with MAFFT v7.526 (Katoh et al., 2005). Finally, phylogenetic trees were built based on the Kimura 2–parameter model (K2 + G + I) using MEGA7 (Kimura, 1980; Kumar et al., 2016). Graphical representation and editing of the phylogenetic trees were performed with Interactive Tree of Life (v3.5.1) (Letunic and Bork, 2016). All the sequences used for phylogenetic reconstructions were downloaded from the NCBI databank using the accession numbers given in Machado et al. (2025) (Table S3 in Supplementary Material). The sequences of *H. caligo* n. sp. UOH-032 generated in this study were deposited in the NCBI under the accession numbers provided in Table S3 in the Supplementary Material.

### Nematode single-gene phylogenetic reconstructions

To reconstruct phylogenetic relationships based on single gene sequences, the following genes/genetic regions were analyzed: the mitochondrial cytochrome c oxidase subunit I (*cox-1*) gene, the internal transcribed spacer (*ITS*) region of the rRNA gene, the calmodulin 1 (*cmd-1*) gene, and the thin filament (F-actin)-associated protein (*unc-87*) gene (Machado et al., 2025). Phylogenetic reconstructions were carried out using the Maximum Likelihood method based on the Kimura 2–parameter (K2 + G + I) model (Kimura, 1980). The trees with the highest log likelihood are shown. The percentage of trees in which the associated taxa clustered together is shown next to the branches. Initial tree(s) for the heuristic search were obtained automatically by applying Neighbor–Join and BioNJ algorithms to a matrix of pairwise distances estimated using the Maximum Composite Likelihood (MCL) approach, and then selecting the tree topology with the superior log likelihood value. A discrete Gamma distribution (+G) was used to model evolutionary rate differences among sites, and the rate variation model allowed for some sites to be evolutionarily invariable (+I). The trees are drawn to scale, with branch lengths measured in terms of substitutions per site. Graphical representation and editing of the phylogenetic trees were performed with Interactive Tree of Life (v3.5.1) (Letunic and Bork, 2016). Sequences of the ITS region of the rRNA gene were extracted from the whole ribosomal RNA operon sequences (see below) using Barrnap 1.2.2. The sequences of the calmodulin 1 (*cmd-1*) gene and the thin filament (F-actin)-associated protein (*unc-87*) gene were obtained by performing a BLAST search using the assembled genome of *H. caligo* n. sp. UOH-032 as the reference and the sequences of *H. safricana* SF281 as queries. All the sequences used for phylogenetic reconstructions were downloaded from the NCBI database using the accession numbers given in the work of Machado et al. (2025) and presented in Table S3 in the Supplementary Material. The sequences of the mitochondrial large subunit ribosomal RNA (*rrnL*) gene, the mitochondrial small subunit ribosomal RNA (*rrnS*) gene, and the D2–D3 expansion segments of the 28S rRNA (*D2D3*) gene were not used due to their poor phylogenetic resolving power (Dhakal et al., 2020; Machado et al., 2021, 2025b; Bhat et al., 2023). However, we deposited the sequences in the NCBI for future studies under the following accession numbers: PX240084 (*rrnL*), PX241222 (*rrnS*), and PX240083 (*D2D3*). In a previous study, Machado et al. (2025) identified the fanconi-associated nuclease 1 (*fan-1*) and the serine/threonine-protein phosphatase 4 regulatory subunit 1 (*ppfr-1*) genes as suitable markers to phylogenetically resolve the different species of the genus *Heterorhabditis*. Given that *H. caligo* n. sp. is closely related to *H. marelatus*, and there are no *fan-1* or *ppfr-1* sequences available for this species due to the lack of laboratory cultures, we did not produce phylogenetic trees using these markers but deposited the sequences in the NCBI for future studies under the following accession numbers: PV892896 (*fan-1*) and PV892897 (*ppfr-1*).

### Nematode whole ribosomal RNA operon phylogenetic reconstructions

The whole ribosomal RNA operon of *H. caligo* n. sp. UOH-032 was obtained by mapping illumina reads to the ribosomal RNA operon sequences of *Caenorhabditis elegans* (NCBI accession number: MN519140) using Bowtie2 v2.5.3 (Langmead and Salzberg, 2012). Mapped reads were then assembled using SPAdes 3.15.5 (Bankevich et al., 2012). The resulting sequences were aligned with the sequences of other *Heterorhabditis* species reported by Machado et al. (2025) using MUSCLE v3.8.31 (Edgar, 2004). The alignment was then used to reconstruct phylogenetic relationships by the Maximum Likelihood method based on the Kimura 2–parameter (K2 + G + I) model (Kimura, 1980). The trees with the highest log likelihood are shown. The percentage of trees in which the associated taxa clustered together is shown next to the branches. Initial tree(s) for the heuristic search were obtained automatically by applying Neighbor–Join and BioNJ algorithms to a matrix of pairwise distances estimated using the MCL approach, and then selecting the tree topology with the superior log likelihood value. A discrete Gamma distribution (+G) was used to model evolutionary rate differences among sites, and the rate variation model allowed for some sites to be evolutionarily invariable (+I). The trees are drawn to scale, with branch lengths measured in terms of substitutions per site. Graphical representation and editing of the phylogenetic trees were performed with Interactive Tree of Life (v3.5.1) (Letunic and Bork, 2016). All the sequences used for phylogenetic reconstructions were downloaded from the NCBI database using the accession numbers given in the work of Machado et al. (2025) and presented in Table S3 in the Supplementary Material. The sequences of *H. caligo* n. sp. UOH-032 generated in this study were deposited in the NCBI under the accession numbers provided also in Table S3 in the Supplementary Material.

## Symbiotic Bacteria Characterization

### Bacteria whole genome sequencing

The symbiotic bacteria associated with *H. caligo* n. sp. UOH-032 were isolated using our standard protocols. Briefly, a drop of hemolymph from *G. mellonella* infected with *H. caligo* n. sp. 24 hr earlier was streaked onto an NBTA agar plate (37 g standard nutrient agar I [Carl Roth, Karlsruhe, Germany], 25 mg bromothymol blue, 1 l distilled water; after sterilization, the medium was cooled to 50°C and supplemented with 4 mL of 1% 2,3,5-triphenyltetrazolium chloride solution). The drop of hemolymph was then spread on the agar using the streak plate method. Petri dishes were sealed with Parafilm. One single colony was further subcultured and used for whole genome sequencing. To this end, gDNA was extracted and purified using Blood and Tissue Kit (Qiagen, Hilden, Germany), following the manufacturer’s instructions. The resulting DNA was used for sequencing using Illumina and Oxford Nanopore technologies. To this end, gDNA was first examined for concentration, purity, and integrity by Nanodrop, Qubit, and 0.35% agarose electrophoresis, respectively. Then, for Illumina-based sequencing, a DNA library was prepared using the TruSeq DNA PCR–Free LT Library Prep (FC–121–3003) kit. Indexed libraries were pooled at equimolar concentrations and sequenced (2 × 150 bp) on an Illumina HiSeq 3000 instrument. Finally, raw Illumina reads were quality trimmed using Trimmomatic 0.39 (Bolger et al., 2014). For ONT sequencing, gDNA was fragmented with gTube to generate DNA fragments of approximately 8 kb. Then, DNA libraries were prepared using the SQK-LSK109 ligation sequencing kit. The products were then purified with magnetic beads. Final products were purified with magnetic beads, and sequenced using the PromethION 48 device. Nanopore sequencing raw data was saved as fast5 format with entire original sequencing signals. Each read corresponds to one fast5 file. Base calling was processed on fast5 file by MinKNOW to generate fastq sequence file. Raw sequences were processed for adapter removal, low-quality reads removal, and short reads removal (Threshold: 2,000 bp). Genomes were assembled using the Illumina and ONT reads using Unicycler (Wick et al., 2017). Minor assembly errors were corrected using Pilon 1.22 (Walker et al., 2014). Completeness and contamination of the assembled genomes were assessed using CheckM v1.2.2 with default parameters (Parks et al., 2015).

### Core genome-based phylogenetic reconstructions and sequence comparisons

To reconstruct whole-genome-based phylogenetic relationships, genomes were first aligned using Roary 3.13.0. Genes to be considered core had to be present in 40% of the genomes with an 80% protein identity. Obtained alignments were used to build phylogenetic trees using FastTree 2.1.10 based on the Generalized Time Reversible Model (GTR). Graphical representation and edition of the phylogenetic trees were performed with Interactive Tree of Life (v3.5.1) (Chevenet et al., 2006; Letunic and Bork, 2016). Digital DNA-DNA hybridization (dDDH) values were used to determine pairwise whole-genome sequence similarities. These values were calculated using the Genome Blast Distance Phylogeny (GBPD) method through the Genome-to-Genome Distance Calculator 2.1 and formula 2 of the Deutsche Sammlung von Mikroorganismen und Zellkulturen (DSMZ) web service (http://ggdc.dsmz.de) using default parameters (Auch et al., 2010a, 2010b; Meier-Kolthoff et al., 2013, 2014). dDDH values of 70% and 79% delimit species and subspecies boundaries, respectively (Wayne et al., 1987; Meier-Kolthoff et al., 2013; Machado et al., 2018). The accession numbers of the sequences used for these analyses are shown in Table S4 in the Supplementary Material.

## Results

### Description

#### Heterorhabditis caligo* n. sp. (Figs. 1–5 and Table 1).

*The specific Latin epithet means “fog” due to the constant morning fog present at the sampling site.

**Figure 1:**
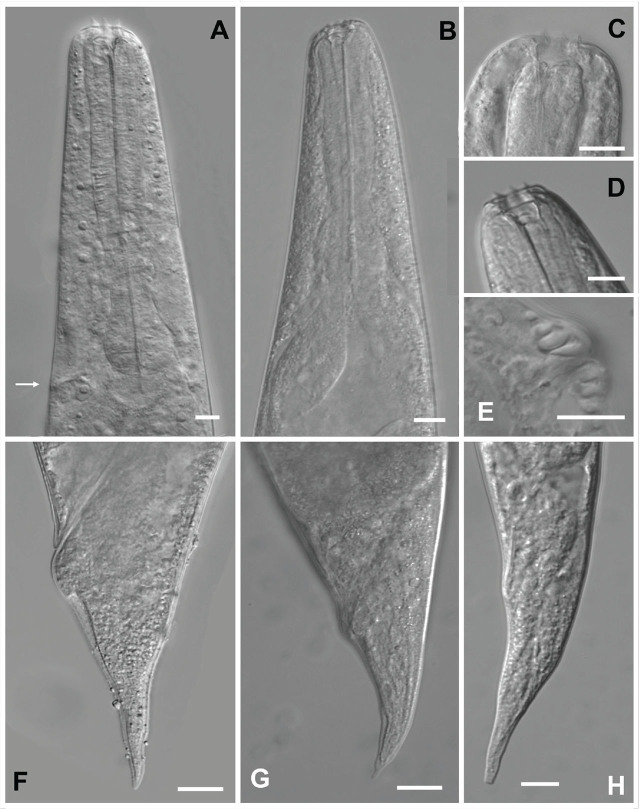
Light microscopy photographs of the hermaphrodite and amphimictic female of *Heterorhabditis caligo* n. sp. (A) anterior region of hermaphrodite, (B) anterior region of the amphimictic female, (C) detail of the head showing the labial papillae of hermaphrodites, (D) detail of the head showing the labial papillae of the amphimictic female, (E) detail of the vulva of the hermaphrodite, (F,G) tail variations of the hermaphrodites, (H) tail of the amphimictic female. Scale bars: A–D and F–H = 20 μm; E = 10 μm.

**Figure 2:**
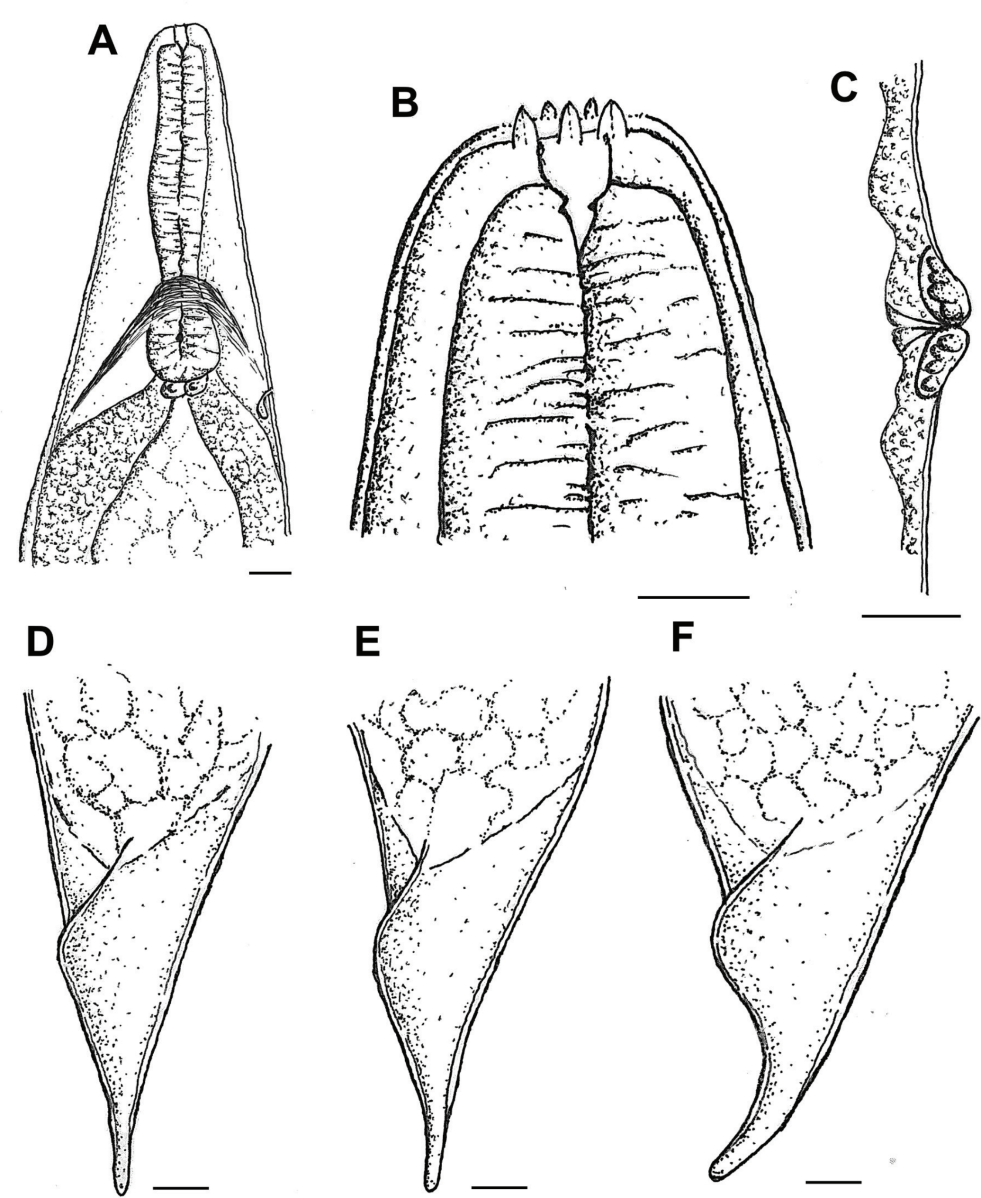
Drawings of the hermaphrodite of *Heterorhabditis caligo* n. sp. (A) anterior region, (B) head region, (C) vulva; (D–F) Tail variations of the hermaphrodites, Scale bars: A–B and D–F = 20 μm; C = 10 μm.

**Figure 3:**
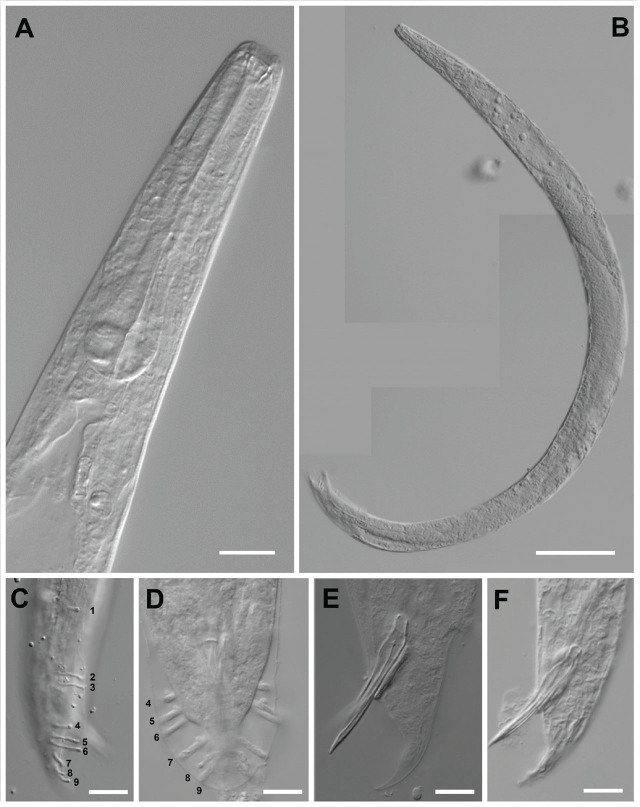
Light microscopy photographs of males of *H. caligo* n. sp. (A) anterior part of the male (B) male *in toto*; (C,D) Lateral and ventral view of papillary arrangement gubernaculum of first-generation male in ventral view; (E,F) tail showing spicula and gubernaculum shapes. *Scale bars:* A = 20 μm; B = 100 μm; C–F = 10 μm.

**Figure 4:**
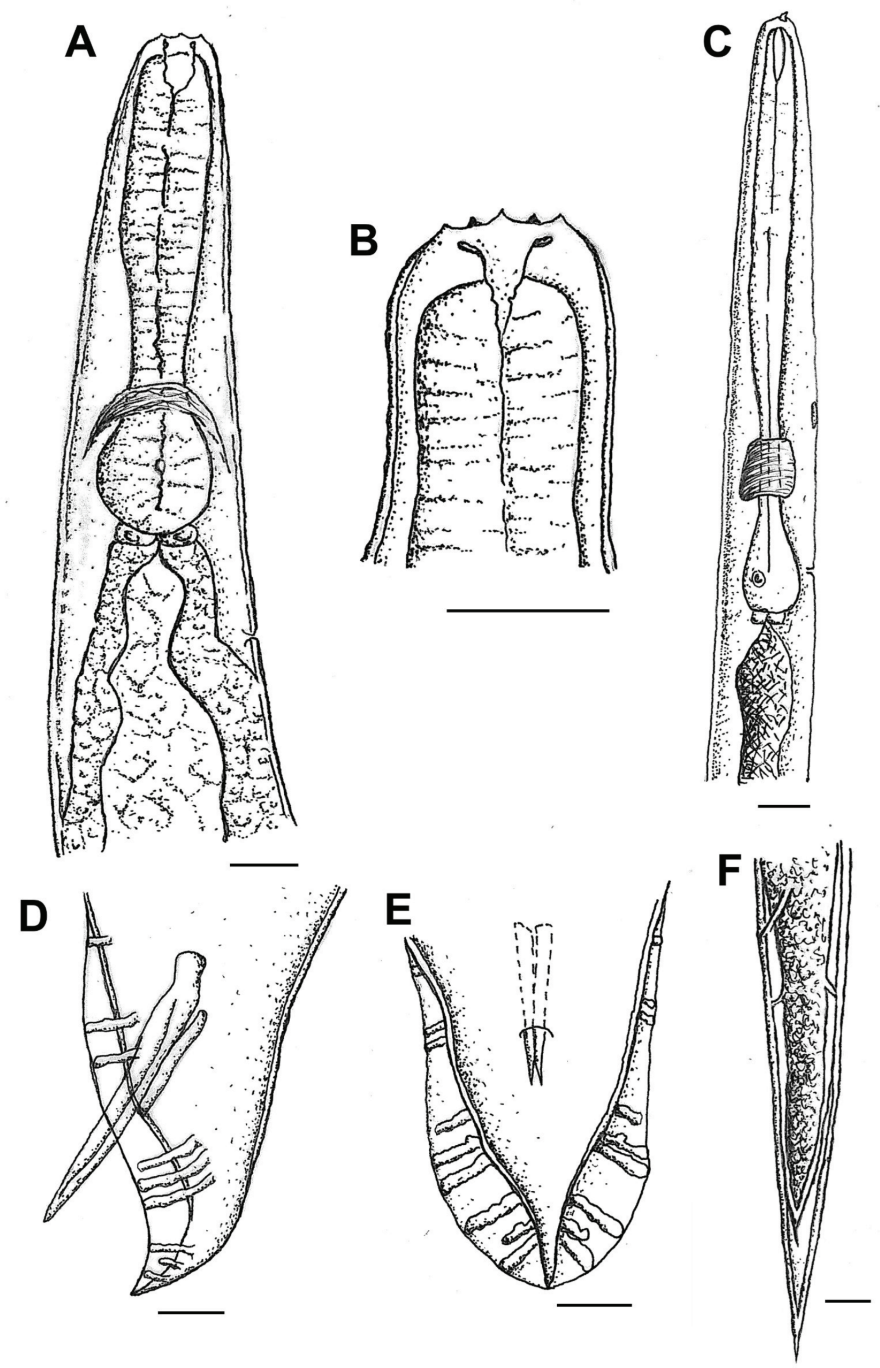
Drawings of male and IJs of *Heterorhabditis caligo* n. sp. (A) anterior region of male; (B) head; (C) anterior region of IJ; (D) posterior portion of male in lateral view, showing spicules, gubernaculum and papillae arrangement; (E) posterior portion of male in ventral view, showing spicules, gubernaculum and papillae arrangement; (F) posterior portion of IJ showing amphids. *Scale bars:* A–B = 20 μm; C–F = 10 μm. IJs, infective juveniles.

**Figure 5:**
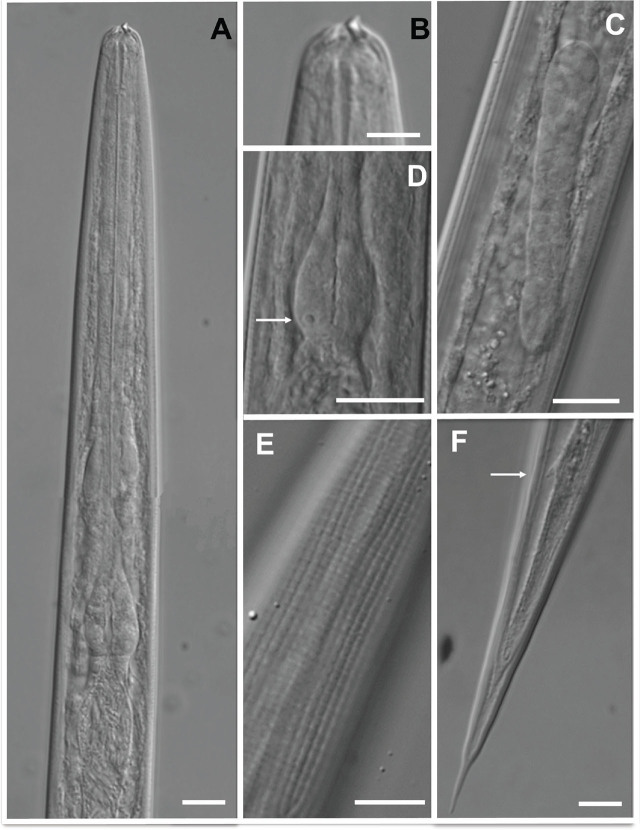
Light microscopy photographs of IJs of *Heterorhabditis caligo* n. sp. (A) anterior part showing bacterial cells in the intestine (arrow), (B) detail of the head showing the dorsal tooth, (C) genital primordium, (D) basal bulb showing subventral gland (arrow), (E) tessellate pattern of the cuticle, (F) tail showing phasmid (arrow). Scale bars = 10 μm. IJs, infective juveniles.

**Table 1:** Morphometrics of *Heterorhabditis caligo* n. sp.

**Character**	**Males**		**Hermaphrodites Paratypes**	**Females Paratypes**	**IJs Paratypes**

	**Holotype**	**Paratypes**			
n	-	20	20	20	20
L	967	1,018 ± 80 (864–1,174)	3,616 ± 602 (2,855–4,950)	2,674 ± 302 (2,002–3,103)	669 ± 43 (568–723)
a	20.1	19 ± 1.08 (16.9–20.8)	14 ± 1.85 (11–18)	15 ± 0.83 (13–17)	27 ± 1.3 (23.7–29.1)
b	8.3	9 ± 0.53 (7.7–9.5)	16 ± 1.36 (13.5–19.19)	16 ± 1.5 (13.1–19.6)	5 ± 0.3 (4.2–5.2)
c	32.2	31 ± 3.45 (24–38.9)	40 ± 7.3 (27.7–55.2)	2 ± 1.8 (2.4–0.13)	6.8 ± 0.5 (5.4–7.7)
c′	1.3	1 ± 0.18 (1.1–1.8)	1.7 ± 0.33 (1.2–2.3)	15 ± 2.2 (11.8–19.6)	6 ± 0.4 (5.6–7.2)
V			46 ± 2.8 (40.3–50)	52 ± 2.47 (45.6–56.8)	
Max. body diam.	48	54 ± 4.1 (45–59)	265 ± 39 (187–336)	183 ± 20.2 (133–210)	25 ± 1.5 (22–28)
Excretory pore	124	131 ± 7.7 (118–146)	239 ± 37.9 (180–310)	193 ± 16.5 (169–224)	119 ± 6.3 (105–128)
Nerve ring	71	74 ± 6.4 (63–92)	156 ± 29 (122–216)	103 ± 15 (77–141)	107 ± 4.2 (95–114)
Pharynx (ES)	117	118 ± 4.2 (112–126)	224 ± 36.6 (175–294)	173 ± 13.7 (148–197)	141 ± 4 (135–150)
Hemizonoid					113 ± 4.0 (106–117)
Testis reflection	128	133 ± 29.1 (85–245)			
Tail length	30	33 ± 4 (25–41)	92 ± 11.9 (71–110)	83 ± 7.3 (74–99)	98 ± 9.7 (84–129)
Tail length without sheath					71 ± 5.1 (60–79)
Anal body diam.	24	23 ± 2.1 (21–27)	55 ± 10.6 (40–75)	42 ± 2.82 (38–50)	16 ± 0.8 (15–18)
Spicule length	51	50 ± 3 (41–52)			
Gubernaculum length	21	21 ± 1.1 (18–23)			
D%	106	112 ± 6.7 (102–129)			84 ± 4.3 (75–91)
E%	413	403 ± 52.3 (367–535)			121 ± 4 (94–135)
SW%	213	214 ± 24.4 (152–248)			
GS%	41	42 ± 3 (35–49)			

All measurements are in μm and in the form of mean ± SD (range).

W = maximum body width; EP = distance from anterior end to excretory pore; NR = distance from anterior end to nerve ring; ES = esophagus length; T = tail length; D% = EP/ES) × 100; E% = (EP/T) × 100.

IJs, infective juveniles.

#### Hermaphroditic females

The measurements of 20 hermaphroditic females are given in Table 1. The body is C-shaped when heat-relaxed, and robust when containing many eggs and embryos. Hatched juveniles are present in older specimens. They are cuticle smooth, with the anterior end tapering anteriorly. The labial region has six prominent lips, each with a terminal labial papilla (Figs. 1A,B and 2B). Cephalic papillae and amphidial apertures are not observed with LM. Stoma is rhabditoid type, with a short cheilostom with a hardly visible refringent rounded cheilorhabdia, gymnostom well-developed, refringent, bar-like rhabdia, and funnel-shaped stegostom surrounded by the pharyngeal collar. The pharynx is with subcylindrical procorpus, metacorpus slightly swollen, narrow isthmus, and basal bulb well developed. Nerve rings surround the isthmus. The excretory pore at the intestine level (Fig. 1A) is always posterior to the basal bulb. The cardia is rounded and protruding into the intestine. The reproductive system is didelphic–amphidelphic. The ovotestes are well developed and reflexed. Uteri are with numerous embryonated eggs. The vagina is short and the vulva is a transverse slit, with no epytigmata and light to moderate protruding lips, close to the mid-body (Fig. 1E). The anal swelling is inconspicuous or moderately developed posteriorly, and the rectum is slender, about 1–2 times the anal body width. The tail conoid has a narrower rounded terminus, lacking a mucron (Figs. 1F,G and 2D–F). Phasmids are inconspicuous.

#### Amphimictic females

The measurements of 20 females are given in Table 1. The body arcuate with general morphology is similar to that of hermaphroditic females. The body is tapering toward the anterior end, which is truncated with six prominent lips bearing labial papillae (Figs. 1B,D). The reproductive system is didelphic–amphidelphic with ovaries well developed, reflexed, vulva slightly protruded (anterior lip = 6 ± 2.8 μm, range 2–11 μm and posterior lip = 7 ± 2.4 μm, range 4–13 μm) with a transverse slit opening with no epitygmata, vagina short, and exudates or copulation plug present in some specimens (6 out of 20). Eggs and embryo are present. Anal swelling is slight to moderate, and the rectum is slender and shorter than in hermaphroditic females (ranging 0.7–1.5 times the anal body width). The tail conoid with a rounded tip lacks a mucron. Phasmids are inconspicuous.

#### Males

The measurements of 20 males are given in Table 1. The body is curved ventrally (open C-shape) or sometimes straight when heat relaxed. The anterior end is truncated (Figs. 3A,B; 4A) and the lip region has six labial papillae. Stoma, with a short cheilostom and refringent rounded cheilorhabdia, has a funnel-shaped stegostom surrounded by the pharyngeal collar (Fig. 4B). The pharynx is with a subcylindrical procorpus, the isthmus is narrower than the metacorpus, and the basal bulb is well developed and spheroid. The nerve ring is located around the isthmus. The excretory pore is always located below the basal bulb (1 basal bulb length). Cardia is conspicuous, rounded, and not protruding into the intestine. The reproductive system is monorchid, with testis reflexed. The vas deferens are well developed. The spicules are also well developed, paired, and colorless with small, quadrangular manubrium set from the lamina by a short calamus. The lamina is almost straight with a single rib and an acute terminus. The gubernaculum is robust, and curved anteriorly in its distal partition in 90% of the specimens. The tail conoid is with an acute tip, ventrally curved, and flanked by the bursa. The bursa peloderan bearis nine pairs of bursal papillae, three precloacal (papillae 1 alone and 2, 3 grouped) and six postcloacal (papillae 4, 5, and 6 grouped and 7, 8, 9 grouped). The fourth and eighth papillae pairs are always shorter within their groups and do not reach the bursal edge (Figs. 3C–F; 4D,E).

#### Infective juveniles

The measurements of 20 IJ are given in Table 1. The body is straight when heat relaxed (Fig. 5A). A sheath (second-stage cuticle) is present. The cuticle is with a tessellate pattern posterior to the lip region in both sheathed and exsheathed specimens (Fig. 5E). The lip region bears the dorsal tooth under the sheath (Figs. 4C and 5A,B). The pharynx is slender, with a cylindrical corpus, a narrower and slender isthmus, and basal bulb pyriform without developed valves. The subventral gland is often seen in 1,000X magnification (Figs. 4C and 5D). A nerve ring surrounds the isthmus. The excretory pore is located anterior to the nerve ring and basal bulb. Hemizonid is visible in 20% of the specimens, always anterior to the excretory pore. Cardia is present, not protruding into the intestine. Symbiotic bacterial cells are clearly distinguishable in the intestine lumen (Fig. 5A). Genital primordium is conspicuous and visible in 400 and 1,000× magnification (about 32 μm long and 6 μm width) (Fig. 5C). Rectum narrow, not clearly discernible in many specimens. Tail conoid with finely pointed terminus. Phasmids often visible in 1,000× magnification (Figs. 4F; 5F).

### Diagnosis and relationships

*Heterorhabditis caligo* n. sp. can be separated from the other species within the *megidis* clade by a combination of the morphological, morphometric, and molecular characteristics. *H. caligo* n. sp. is morphologically and morphometrically similar to *H. marelatus* with the following differences: In males, the fourth and eighth pairs of the bursal papillae are shorter and do not reach the edge of the bursa in *H. caligo* n. sp. (Figs. 3D and 4E), whereas all the papillae in *H. marelatus* reach the edge of the bursa. The excretory pore of amphimictic females of *H. caligo* n. sp. is located more posteriorly than in those of *H. marelatus* 193 (169–224) μm vs. 157 (139–178) μm, respectively. The pharynx of *H. caligo* n. sp. is longer than that of *H. marelatus* 173 (148–197) μm vs. 144 (129–164) μm, and the tale of the former is longer than that of *H. marelatus* 83 (74–99) μm vs. 67 (55–81). The IJs of *H. caligo* n. sp. are similar in length but differ from *H. marelatus* by the longer pharynx, 141 (135–150) μm vs. 133 (121–138) μm, and the position of the excretory pore from the anterior end, 119 (105–128) μm vs. 102 (81–113) μm. In *H. caligo* n. sp., the hemizonid is always located anterior to the excretory pore, whereas in *H. marelatus* the hemizonid is usually located just posterior to the excretory pore.

The males of *H. caligo* n. sp. can be separated from males of *H. megidis* by the shape of the bursa (peloderan vs. pseudopeloderan), and the lack of a fine tip extending beyond the bursal membrane present in the latter, shorter distance from the anterior end to the excretory pore 119 (105–128) μm vs. 156 (139–176) μm. The IJs of *H. caligo* n. sp. are smaller compared with *H. megidis* 667 (568–723) μm vs. 768 (736–800) μm), and have a shorter distance from the head to the excretory pore 119 (105–128) μm vs. 131 (123–142) μm.

Males of *H. caligo* n. sp. have excretory pores more posteriorly located than the males of *H. downesi* 131 (118–146) μm vs. 89 (86–91) μm, respectively, and a longer pharynx 118 (112–126) μm vs. 101 (97–106) μm, respectively. Hermaphroditic females of *H. caligo* n. sp. can be differentiated from hermaphroditic females of *H. downesi* by the shape of their tails (conoid with rounded tip lacking a mucron vs blunt and mucronate). IJs of *H. caligo* n. sp. have longer tails than the IJs of *H. downesi* 98 (84–129) μm vs. 68 (62–74) μm; they differ in the a ratio 27 (24–29) vs. 35 (29–42), respectively, and c ratio 6.8 (5.4–7.7) vs. 9.5 (8.5–10.5) and lack the spike-like tip present in the IJs of *H. downesi*.

The males of *H. caligo* n. sp. can be differentiated from those of *H. safricana* by their GS% 42 (35–49) vs. 53.9 (43.3–61.7), respectively. The amphimictic females of *H. caligo* n. sp. have a longer distance from the anterior end to the excretory pore compared with *H. safricana* females at 193 (169–224) μm vs. 171 (151–196) μm.

The amphimictic females of *H. caligo* n. sp. have a longer distance from the anterior end to the excretory pore compared with *H. atacamensis* females at 193 (169–224) μm vs. 161 (154–182) μm, and pharynx length 173 (148–197) vs. 150 (129–167). IJs of *H. caligo* n. sp. are longer compared with the IJs from *H. atacamensis*, 667 (568–723) μm vs. 611 (578–666) μm, but have a shorter E% 121 (94–135) vs. 165 (149–182).

*Heterorhabditis caligo* n. sp. is morphologically and morphometrically similar in all stages compared with *H. zealandica* but the IJs of the former lack the spine-like terminus present in the latter. In males the disposition of the bursal papillae varies between both species: in the case of *H. caligo* n. sp., the terminal papillae form a clear cluster of three pairs (seventh, eighth and ninth), whereas in *H. zealandica* the seventh pair is somewhat closer to the sixth papillae rather than the eighth. Also, the fourth bursal papillae of *H. caligo* n. sp. do not reach the edge as in *H. zealandica*.

### Type host and locality

The host of this nematode in nature is unknown. *H. caligo* n. sp. was isolated, using the *Galleria* baiting method, from a coastal dune between the Petrel lagoon and the seashore, in Pichilemu (central Chile) (−34.379151, −71.995739). The typical vegetation of these dunes comprises *Ammophila arenaria* (Poales: Poaceae), *Ambrosia chamissonis* (Asterales: Asteraceae), and *Carpobrotus chilensis* (Caryophyllales: Aizoaceae). Although the dunes were relatively small, we successfully isolated this new species, which co-occurred with a population of *Steinernema australe.* Pichilemu and its coastal lagoon are characterized by a Mediterranean climate with a mean yearly temperature of 13.9°C. The region experiences winter-dominant precipitation. These coastal wetlands are ecologically important but increasingly vulnerable, as dwindling winter rainfall causes water levels to drop, severing seasonal connections to the Pacific Ocean and affecting the brackish nature of the lagoons.

### Type material

A slide containing the male holotype and Eppendorf tubes containing 50 hermaphrodites, 50 females, and 50 males fixed in TAF were deposited in the National Museum of Natural Sciences (Madrid, Spain). The rest of the specimens, 15 males and 15 females, remain in the Laboratory of Nematology at the Universidad de O’Higgins, Chile. As required by the International Commission on Zoological Nomenclature, the ZooBank registration number for the new Linnaean binomials is LSID urn:lsid:zoobank.org:pub:446F3205-AD35-4655-8DB9-AAB86E99A6FA

### Molecular Characterization and Phylogenetic Analysis

#### Nematode phylogenetic relationship reconstructions

**Figure 6:**
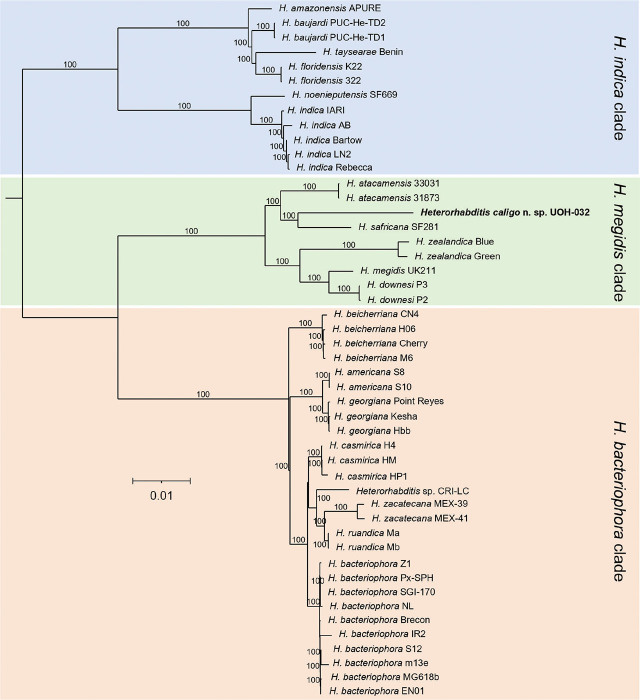
Approximately-maximum-likelihood phylogenetic trees reconstructed from concatenated sequences of orthogroups of different *Heterorhabditis* species. A total of 4,600 single-copy orthogroups, comprising 1,846,787 amino acid positions, were analyzed. Numbers at the nodes represent bootstrap values based on 500 replications. Bars represent average nucleotide substitutions per sequence position. *Heterorhabditis mexicana* from the *indica* clade and *H. marelatus* from the *megidis* clade could not be included in the analyses due to lack of laboratory cultures.

**Figure 7:**
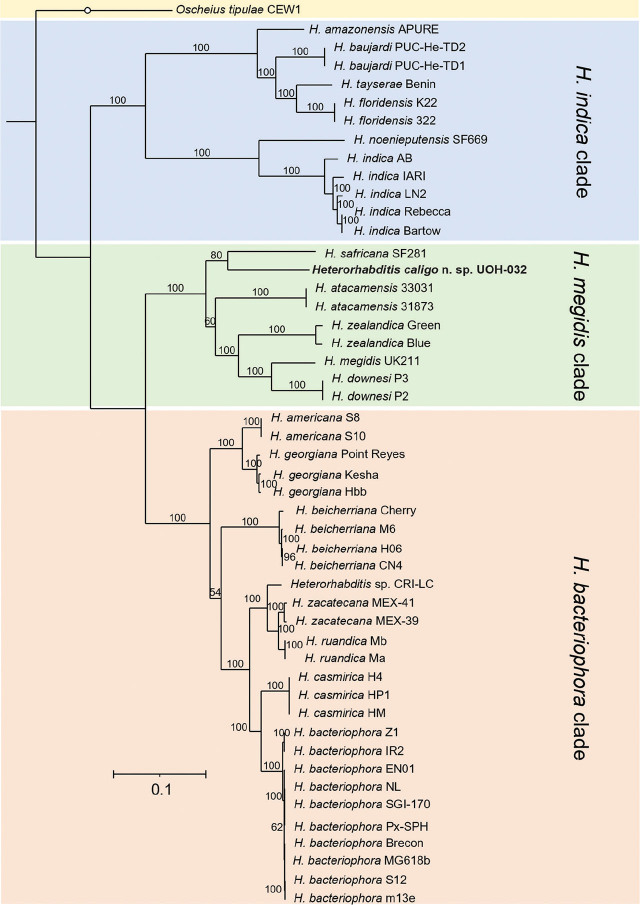
Maximum-likelihood phylogenetic tree reconstructed from concatenated sequences of the following protein-coding genes: cytochrome c oxidase, cytochrome b, and NADH dehydrogenase of the mitochondrial genomes of different *Heterorhabditis* species. A total of 9,494 nucleotide positions were analyzed. The genes were concatenated in the following order: *cob*, *cox-1*, *cox-2*, *cox-3*, *nad-1*, *nad-2*, *nad-3*, *nad-4*, *nad-4l*, *nad-5*, and *nad-6*. Accession numbers of the concatenated sequences used for the analyses are shown in Table S3 in the Supplementary Material. Numbers at nodes represent bootstrap values based on 500 replications. Bars represent average nucleotide substitutions per sequence position. *Heterorhabditis mexicana* from the *indica* clade and *H. marelatus* from the *megidis* clade could not be included in the analyses due to lack of laboratory cultures.

**Figure 8:**
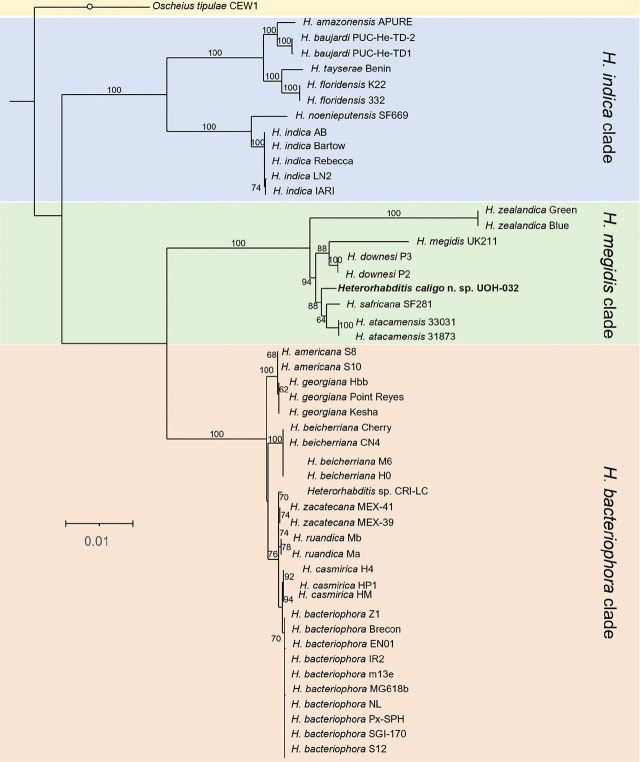
Maximum-likelihood phylogenetic tree reconstructed from the whole ribosomal RNA operons of different *Heterorhabditis* species. A total of 5,927 nucleotide positions were analyzed. Accession numbers of the sequences used for the analyses are shown in Table S3 in the Supplementary Material. Numbers at nodes represent bootstrap values based on 500 replications. Bars represent average nucleotide substitutions per sequence position. *Heterorhabditis mexicana* from the *indica* clade and *H. marelatus* from the *megidis* clade could not be included in the analyses due to lack of laboratory cultures.

**Figure 9:**
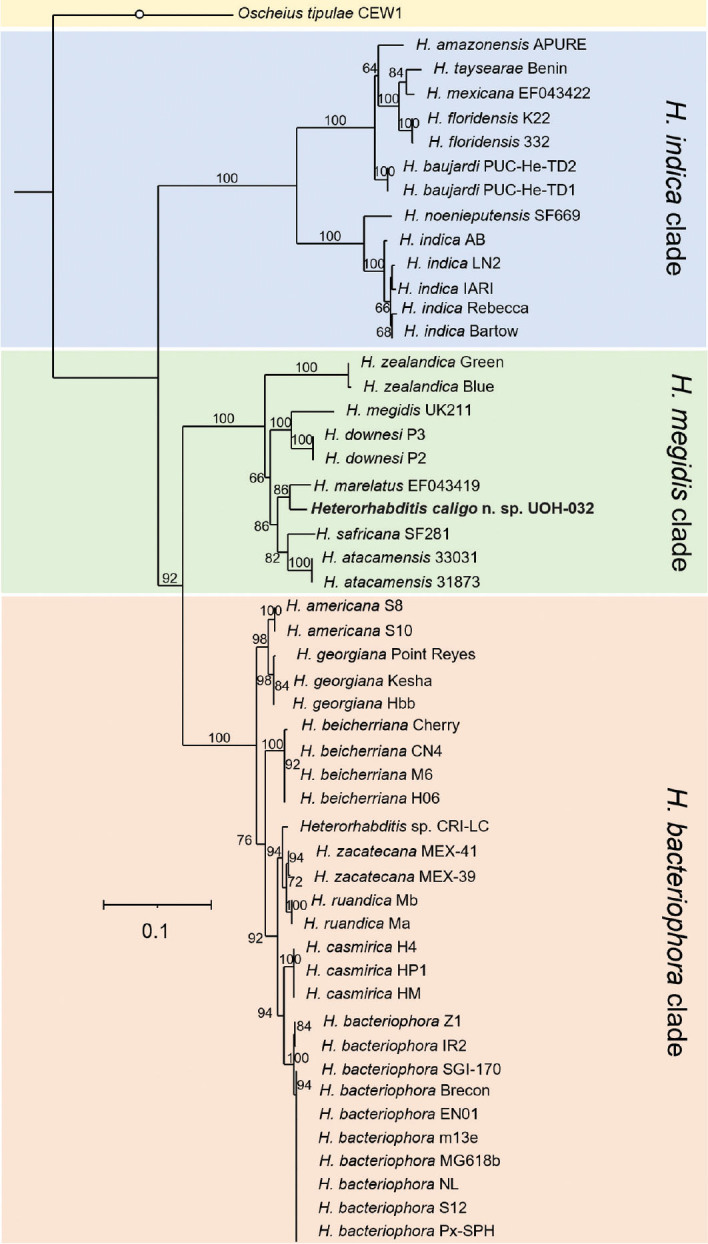
Maximum aphylogenetic tree reconstructed from the concatenated sequences of the ITS region of the rRNA gene and the cytochrome c oxidase subunit I (*cox-1*) gene of different *Heterorhabditis* species. A total of 1,482 nucleotide positions were analyzed. The ITS sequences of *H. marelatus* and *H. mexicana* were obtained from the NCBI using the accession numbers AY321479 and EF043444, respectively. The ITS sequences of all the other isolates were extracted from whole ribosomal RNA operons. These sequences were then trimmed to obtain sequences that cover the region flanked by the commonly used primers TW81 and AW28. The *cox-1* sequences of *H. marelatus* and *H. mexicana* were obtained from the NCBI using the accession numbers EF043419 and EF043422, respectively. The sequences of all the other isolates were extracted from whole mitochondrial genomes. These sequences were then trimmed to obtain sequences that cover the region flanked by the commonly used primers HCF and HCR. Accession numbers of the nucleotide sequences used for the analyses are shown in Table S3 in the Supplementary Material. Numbers at nodes represent bootstrap values based on 500 replications. Bars represent average nucleotide substitutions per sequence position. ITS, internal transcribed spacer; NCBI, National Center for Biotechnology Information.

**Figure 10:**
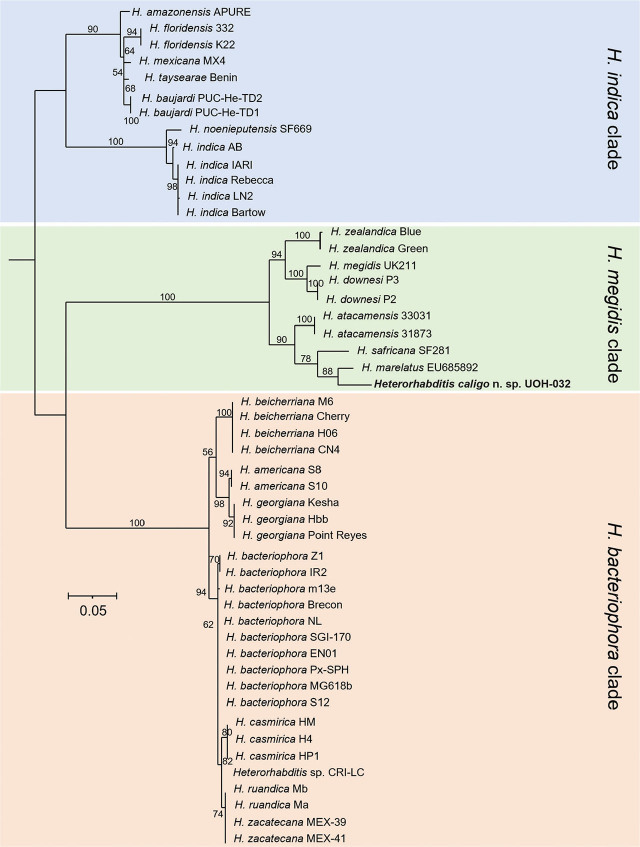
Maximum-likelihood phylogenetic tree reconstructed from the nucleotide sequences of the calmodulin 1 (*cmd-1*) gene. A total of 738 nucleotide positions were analyzed. Accession numbers of the nucleotide sequences used for the analyses are shown in Table S3 in the Supplementary Material. Numbers at nodes represent bootstrap values based on 500 replications. Bars represent average nucleotide substitutions per sequence position. Trees were rooted at the midpoint.

**Figure 11:**
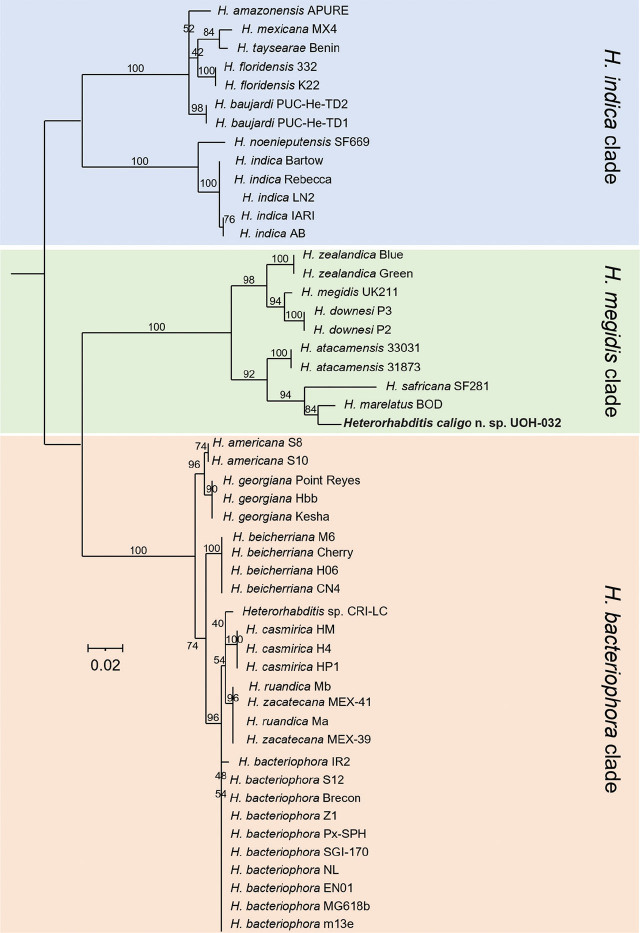
Maximum-likelihood phylogenetic tree reconstructed from the nucleotide sequences of the thin filament F-actin-associated protein (*unc-87*) gene. A total of 465 nucleotide positions were analyzed. Accession numbers of the nucleotide sequences used for the analyses are shown in Table S3 in the Supplementary Material. Numbers at nodes represent bootstrap values based on 500 replications. Bars represent average nucleotide substitutions per sequence position. Trees were rooted at the midpoint.

Whole nuclear and mitochondrial genome, and whole ribosomal operon-based trees show that *H. caligo* n. sp. belongs to the *megidis* clade and is closely related to *H. safricana* (Figs. 6–8). Although these phylogenetic reconstructions enable robust species discrimination and inference of phylogenetic relationships in the genus *Heterorhabditis*, they are insufficient to show that *H. caligo* n. sp. is a novel species due to the lack of available sequences of *H. marelatus*, a member of the *megidis* clade, which is closely related to *H. safricana* and *H. caligo* n. sp. As we have not yet been able to obtain whole genome sequences of *H. marelatus* because there are no *H. marelatus* specimens in laboratory cultures, we reconstructed phylogenetic relationships using additional available sequences. Phylogenetic trees using individual or concatenated sequences of the ITS region of the rRNA gene, the cytochrome c oxidase subunit I (*cox-1*) gene, the calmodulin 1 (*cmd-1*) gene, and thin filament F-actin-associated protein (*unc-87*) gene show often a clear separation between *H. caligo* n. sp. and its sister species *H. marelatus*, *H. safricana*, and *H. atacamensis* (Figs. 9–11; Figures S1 and S2 in the Supplementary Material). At the nucleotide level, *H. caligo* n. sp. shares no more than 93% sequence identity in the cytochrome c oxidase subunit I (*cox-1*) gene, 98.9% in the ITS region of the rRNA gene, 92.5% in the calmodulin 1 (*cmd-1*) gene, and 97.2% in the thin filament F-actin-associated protein (*unc-87*) gene with its sister species *H. marelatus*, *H. safricana*, and *H. atacamensis* (Table S5 in the Supplementary Material). Different isolates of the same species share more than 98.7% in the sequences of the cytochrome c oxidase subunit I (*cox-1*) gene, more than 99.8% in the sequences of the ITS region of the rRNA gene, more than 99.1% in the sequences of the calmodulin 1 (*cmd-1*) gene, and more than 99.5% in the sequences of the thin filament F-actin-associated protein (*unc-87*). Hence, *H. caligo* n. sp. represents a novel phylogenetically distinct species.

### Bacterial symbionts

**Figure 12:**
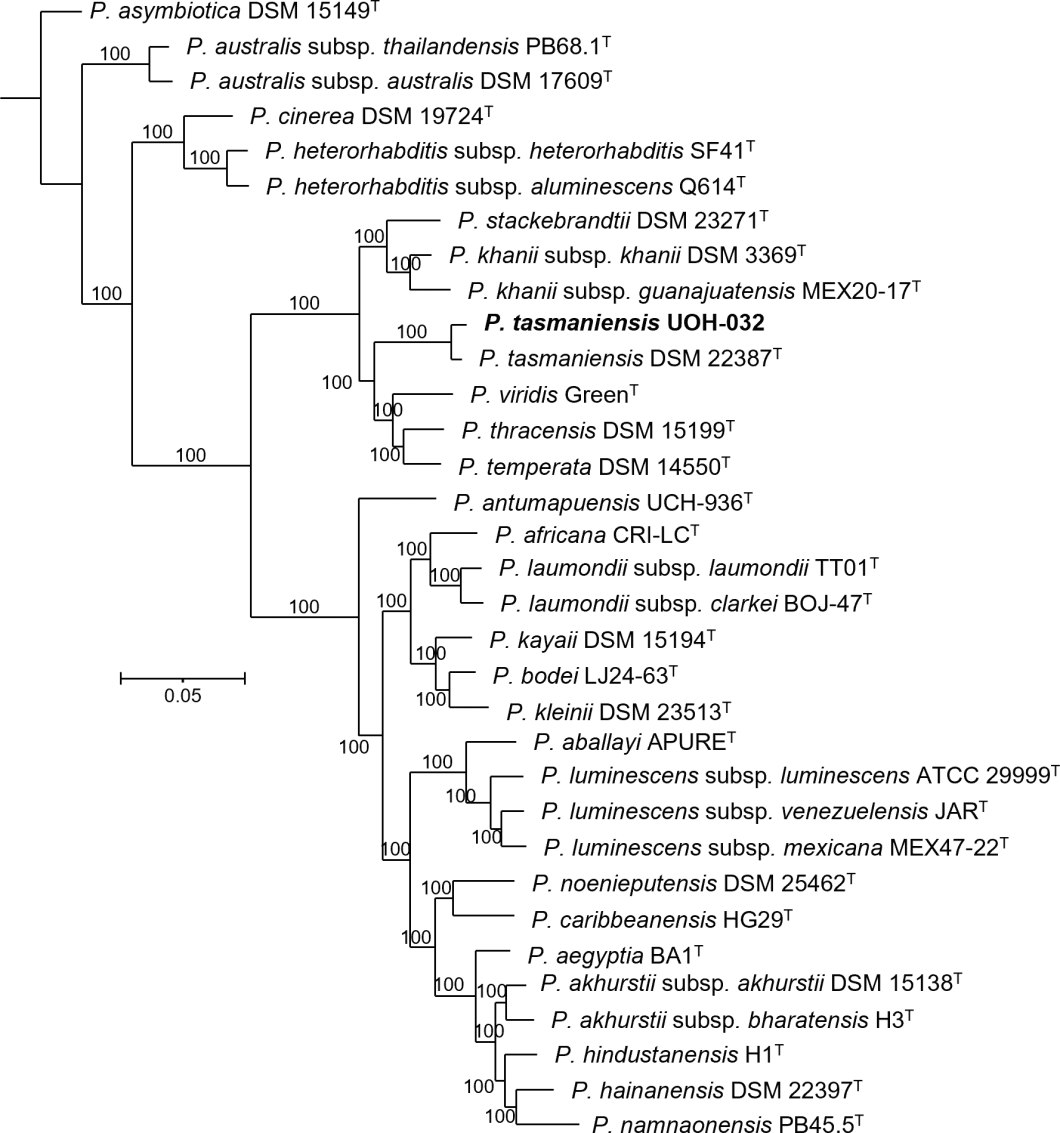
Phylogenetic reconstruction based on core genome sequences of Photorhabdus type strains with validly published names. A total of 3,526,609 nucleotide positions (3,455 core genes) were used in the analyses. Numbers at the nodes represent SH-like branch supports. Bar represents 0.05 nucleotide substitutions per sequence position. Accession numbers of the genome sequences used for the reconstruction are shown in Table S4 in the Supplementary Material.

Phylogenetic reconstructions based on core genome sequences and sequence comparisons show that the bacterial symbiont isolated from *H. caligo* n. sp. belongs to the species *P. tasmaniensis* (Fig. 12). The dDDH scores between DSM 22387^T^, the type strain of the species *P. tasmaniensis*, and strain UOH-032, isolated from *H. caligo* n. sp., is 85.3%, which is above the 70% and 79% thresholds that delimit prokaryotic species and subspecies, respectively, showing that they are conspecific (Table S6 in the Supplementary Material). The intraspecific dDDH values in the genus *Photorhabdus* are greater than 87%, and often greater than 97% (Machado et al., 2018, 2021).

### Data availability statement

The sequences of *H. caligo* n. sp. UOH-032 were deposited in the NCBI database under the accession numbers shown in Table S3 in the Supplementary Material. In addition, the sequences of the *fan-1*, the *ppfr-1*, *rrnL*, *rrnS*, and *D2D3* genes were deposited under accession numbers PV892896, PV892897, PX240084, PX241222, and PX240083, respectively.

## Discussion

The discovery of *Heterorhabditis caligo* n. sp. from the coastal dunes of Pichilemu represents a significant addition to the known diversity of EPNs in South America, particularly within Chile’s Mediterranean climate zone. The species’ occurrence in sandy coastal habitats suggests ecological adaptations to arid, salt-influenced environments that may distinguish it from other members of the *megidis* group typically found in more mesic conditions. The association with *Photorhabdus tasmaniensis* as its bacterial symbiont further emphasizes the complex co-evolutionary relationships within these nematode–bacteria partnerships, particularly noteworthy given that this bacterial species was originally described from Australia, suggesting either broader geographic distribution patterns or convergent symbiotic associations across distant biogeographic regions.

The discovery of *H. caligo* n. sp. highlights the ongoing need for comprehensive taxonomic characterization within the *Heterorhabditis* group, particularly regarding its intraspecific relationships. While, due to the availability of living specimens, the intraspecific molecular relationships of most of the species have been studied in the past, this information is still lacking for some species such as *H. marelatus*, *H. safricana*, and the newly described species (Machado et al., 2025). This is particularly relevant given the relatively high sequence similarity values of some taxonomically relevant gene markers.

The distinctive morphological characters, particularly the shortened fourth and eighth bursal papillae in males and the posterior positioning of the excretory pore in females, provide reliable diagnostic features. However, the absence of laboratory cultures for several closely related species, particularly *H. marelatus*, limits comprehensive morphological analysis and underscores the critical need for establishing culture collections of EPNs to support both taxonomic research and biological control applications.

## Supplementary Materials

### Supplementary Tables

**Table S1:** Features and assemble statistics of the nematode genomes generated and/or used in this study.

**Species**	**Strain designation(s)**	**L50**	**N50**	**L90**	**N90**	**Genome size (bp)**	**GC content (%)**
*H. amazonensis*	APURE	185	94744	692	21806	65101680	33.72
*H. americana*	S10	432	48365	1815	6421	72782968	33.07
	S8	635	33153	2886	3735	72234711	33.27
*H. atacamensis*	31873	212	82969	940	12449	67224907	32.98
	33031	218	81903	981	11470	67409700	33.09
	Brecon	585	35417	2251	6475	69889708	32.85
	EN01	593	35153	2635	4057	71959660	33.02
	IR2	422	50708	1521	10601	70203201	32.90
	m13e	787	27231	3030	4697	72585993	33.08
*H. bacteriophora*	MG618b	639	33083	2791	4049	71868631	33.02
	NL	424	49858	1597	9515	71077392	33.14
	S12	594	35368	2637	4102	71872306	33.00
	SGI-170	415	50445	1801	5833	73250772	33.13
	Px-SPH	396	53254	1778	5674	73984962	33.22
	Z1	383	54630	1655	6479	73293000	33.13
*H. baujardi*	PUC-He-TD1	166	102718	632	23255	64662943	33.73
	PUC-He-TD2	210	86004	814	15671	66175497	33.60
	Cherry	387	53208	1595	7903	71960759	32.97
*H. beicherriana*	CN4	740	28317	3011	4301	70797395	32.83
	H06	586	35927	2488	4871	70742037	32.82
	M6	378	55114	1600	7533	72276565	33.06

***H. caligo* n. sp.**	**UOH-032**	**216**	**90000**	**991**	**11663**	**72194284**	**33.78**

	H4	410	52252	1770	6413	73080526	33.16
*H. casmirica*	HM	412	51774	1771	6469	73036779	33.15
	HP1	496	40787	1808	9330	70153441	32.94
*H. downesi*	P2, pur2	582	33455	2189	6602	66295249	33.33
	P3, pur3	344	53355	1310	11663	65144250	33.18
*H. floridensis*	332	195	92043	712	21459	64561023	33.74
	K22	190	94213	711	21075	64790060	33.79
	Hbb	617	34453	2713	4209	71543875	32.94
*H. georgiana*	Kesha	432	49201	1880	5965	72925328	33.06
	Point Reyes	425	49428	1803	6471	72527224	33.03
	AB, HeM	225	79854	917	12606	65680349	33.80
	Bartow	247	73385	1017	10433	66304658	33.86
*H. indica*	IARI, IARI-EPN-Hms1	73	234422	298	60148	67546827	32.34
	LN2	244	74414	1018	10332	66427391	33.91
	Rebecca	223	81088	916	11868	65936969	33.83
*H. megidis*	UK211	234	77932	1025	11317	66720551	33.23
*H. noenieputensis*	SF669	272	66412	1186	7769	66740923	34.02
*H. ruandica*	Rw18_M-Hr1a	456	46969	2072	4705	73994590	32.80
	Rw18_M-Hr1b	451	48699	2058	5143	74807992	33.81
*H. safricana*	SF281	181	92924	847	12279	66942788	33.06
*H. taysearae*	Benin	235	78313	928	12299	66991707	34.04
*H. zacatecana*	MEX-39	450	46782	1866	6629	72489781	33.01
	MEX-41	611	34263	2558	4886	71022245	32.85
*H. zealandica*	Blue, SF41	283	66513	1280	8325	67614449	33.11
	Green, MJ2C	216	83151	979	10895	67060566	33.09
*Heterorhabditis* sp.	CRI-LC	410	50459	1639	7686	72017000	32.97

**Table S2:** Features and annotation statistics of the nematode genomes generated and/or used in this study.

**Species**	**Strain designation(s)**	**BUSCO Genome assembly**	**Number of proteins**	**BUSCO proteins**
*H. amazonensis*	APURE	83	11725	82
*H. americana*	S10	80	20149	81
	S8	80	18366	80
*H. atacamensis*	31873	82	13256	75
	33031	82	13239	81
	Brecon	81	15525	71
	EN01	80	19242	75
	IR2	81	19233	77
	m13e	80	19084	77
*H. bacteriophora*	MG618b	80	19127	81
	NL	80	15490	81
	S12	80	19200	81
	SGI-170	81	21150	83
	Px-SPH	81	24383	76
	Z1	81	21949	75
*H. baujardi*	PUC-He-TD1	83	11561	76
	PUC-He-TD2	83	11648	74
	Cherry	82	20528	80
*H. beicherriana*	CN4	80	13778	77
	H06	82	17382	81
	M6	82	25282	72

***H. caligo* n. sp.**	**UOH-032**	**82**	**16223**	**80**

*H. casmirica*	H4	81	18294	72
	HM	80	18376	76
	HP1	80	15124	71
*H. downesi*	P2, pur2	80	12235	83
	P3, pur3	81	12257	83
*H. floridensis*	332	83	11541	82
	K22	83	11587	76
	Hbb	80	19156	81
*H. georgiana*	Kesha	80	23309	76
	Point Reyes	81	20357	82
	AB, HeM	82	12823	82
	Bartow	82	13059	85
*H. indica*	IARI, IARI-EPN-Hms1	84	11844	81
	LN2	82	13088	80
	Rebecca	83	12933	81
*H. megidis*	UK211	82	13224	80
*H. noenieputensis*	SF669	82	13418	75
*H. ruandica*	Rw18_M-Hr1a	80	18441	81
	Rw18_M-Hr1b	81	18274	79
*H. safricana*	SF281	82	12947	75
*H. taysearae*	Benin	83	20647	70
*H. zacatecana*	MEX-39	80	24958	76
	MEX-41	81	24402	81
*H. zealandica*	Blue, SF41	82	21057	81
	Green, MJ2C	83	20417	82
*Heterorhabditis* sp.	CRI-LC	81	19815	81

**Table S3:** National Center for Biotechnology Information (NCBI) databank accession numbers of the nematode sequences used for phylogenetic reconstructions.

**Species**	**Strain designation(s)**	**Whole rRNA Operon**	**cob**	**Partial**	**Complete**											
				***cox-1***	***cox-1***	***cox-2***	***cox-3***	***nad-1***	***nad-2***	***nad-3***	***nad-4***	***nad-4I***	***nad-5***	***nad-6***	***unc-87***	***cmd-1***
*H. amazonensis*	APURE	PQ345856	PQ428464	PQ368640	PQ341126	PQ428511	PQ428558	PQ428605	PQ428652	PQ428699	PQ367523	PQ428746	PQ428793	PQ428840	PQ367570	PQ367617
*H. americana*	S10	PQ345891	PQ428499	PQ368684	PQ341161	PQ428546	PQ428593	PQ428640	PQ428687	PQ428734	PQ367558	PQ428781	PQ428828	PQ428875	PQ367605	PQ367652
	S8	PQ345893	PQ428501	PQ368683	PQ341163	PQ428548	PQ428595	PQ428642	PQ428689	PQ428736	PQ367560	PQ428783	PQ428830	PQ428877	PQ367607	PQ367654
*H. atacamensis*	31873	PQ345852	PQ428460	PQ368641	PQ341122	PQ428507	PQ428554	PQ428601	PQ428648	PQ428695	PQ367519	PQ428742	PQ428789	PQ428836	PQ367566	PQ367613
	33031	PQ345853	PQ428461	PQ368642	PQ341123	PQ428508	PQ428555	PQ428602	PQ428649	PQ428696	PQ367520	PQ428743	PQ428790	PQ428837	PQ367567	PQ367614
	Brecon	PQ345860	PQ428468	PQ368643	PQ341130	PQ428515	PQ428562	PQ428609	PQ428656	PQ428703	PQ367527	PQ428750	PQ428797	PQ428844	PQ367574	PQ367621
	EN01	PQ345864	PQ428472	PQ368644	PQ341134	PQ428519	PQ428566	PQ428613	PQ428660	PQ428707	PQ367531	PQ428754	PQ428801	PQ428848	PQ367578	PQ367625
	IR2	PQ345872	PQ428480	PQ368645	PQ341142	PQ428527	PQ428574	PQ428621	PQ428668	PQ428715	PQ367539	PQ428762	PQ428809	PQ428856	PQ367586	PQ367633
*H. bacteriophora*	m13e, TT01	PQ345876	PQ428484	PQ368646	PQ341146	PQ428531	PQ428578	PQ428625	PQ428672	PQ428719	PQ367543	PQ428766	PQ428813	PQ428860	PQ367590	PQ367637
	MG618b	PQ345882	PQ428490	PQ368647	PQ341152	PQ428537	PQ428584	PQ428631	PQ428678	PQ428725	PQ367549	PQ428772	PQ428819	PQ428866	PQ367596	PQ367643
	NL	PQ345883	PQ428491	PQ368648	PQ341153	PQ428538	PQ428585	PQ428632	PQ428679	PQ428726	PQ367550	PQ428773	PQ428820	PQ428867	PQ367597	PQ367644
	S12	PQ345892	PQ428500	PQ368650	PQ341162	PQ428547	PQ428594	PQ428641	PQ428688	PQ428735	PQ367559	PQ428782	PQ428829	PQ428876	PQ367606	PQ367653
	SGI-170, SGI170	PQ345896	PQ428504	PQ368651	PQ341166	PQ428551	PQ428598	PQ428645	PQ428692	PQ428739	PQ367563	PQ428786	PQ428833	PQ428880	PQ367610	PQ367657
	Px-SPH, PxSPH	PQ345889	PQ428497	PQ368649	PQ341159	PQ428544	PQ428591	PQ428638	PQ428685	PQ428732	PQ367556	PQ428779	PQ428826	PQ428873	PQ367603	PQ367650
	Z1	PQ345898	PQ428506	PQ368652	PQ341168	PQ428553	PQ428600	PQ428647	PQ428694	PQ428741	PQ367565	PQ428788	PQ428835	PQ428882	PQ367612	PQ367659
*H. baujardi*	PUC-He-TD1	PQ345887	PQ428495	PQ368653	PQ341157	PQ428542	PQ428589	PQ428636	PQ428683	PQ428730	PQ367554	PQ428777	PQ428824	PQ428871	PQ367601	PQ367648
	PUC-He-TD2	PQ345888	PQ428496	PQ368654	PQ341158	PQ428543	PQ428590	PQ428637	PQ428684	PQ428731	PQ367555	PQ428778	PQ428825	PQ428872	PQ367602	PQ367649
	Cherry	PQ345861	PQ428469	PQ368655	PQ341131	PQ428516	PQ428563	PQ428610	PQ428657	PQ428704	PQ367528	PQ428751	PQ428798	PQ428845	PQ367575	PQ367622
*H. beicherriana*	CN4	PQ345862	PQ428470	PQ368656	PQ341132	PQ428517	PQ428564	PQ428611	PQ428658	PQ428705	PQ367529	PQ428752	PQ428799	PQ428846	PQ367576	PQ367623
	H06	PQ345866	PQ428474	PQ368657	PQ341136	PQ428521	PQ428568	PQ428615	PQ428662	PQ428709	PQ367533	PQ428756	PQ428803	PQ428850	PQ367580	PQ367627
	M6	PQ345877	PQ428485	PQ368658	PQ341147	PQ428532	PQ428579	PQ428626	PQ428673	PQ428720	PQ367544	PQ428767	PQ428814	PQ428861	PQ367591	PQ367638
***H. caligo* n. sp.**	**UOH-032**	**PV871102**	**PV873291**	**PV873292**	**PV873292**	**PV873293**	**PV873294**	**PV873295**	**PV873296**	**PV873297**	**PV873298**	**PV873300**	**PV873299**	**PV873301**	**PV892898**	**PV892895**
	H4	PQ345867	PQ428475	PQ368659	PQ341137	PQ428522	PQ428569	PQ428616	PQ428663	PQ428710	PQ367534	PQ428757	PQ428804	PQ428851	PQ367581	PQ367628
*H. casmirica*	HM	PQ345869	PQ428477	PQ368660	PQ341139	PQ428524	PQ428571	PQ428618	PQ428665	PQ428712	PQ367536	PQ428759	PQ428806	PQ428853	PQ367583	PQ367630
	HP1	PQ345870	PQ428478	PQ368661	PQ341140	PQ428525	PQ428572	PQ428619	PQ428666	PQ428713	PQ367537	PQ428760	PQ428807	PQ428854	PQ367584	PQ367631
*H. downesi*	P2, pur2	PQ345884	PQ428492	PQ368662	PQ341154	PQ428539	PQ428586	PQ428633	PQ428680	PQ428727	PQ367551	PQ428774	PQ428821	PQ428868	PQ367598	PQ367645
	P3, pur3	PQ345885	PQ428493	PQ368663	PQ341155	PQ428540	PQ428587	PQ428634	PQ428681	PQ428728	PQ367552	PQ428775	PQ428822	PQ428869	PQ367599	PQ367646
*H. floridensis*	332	PQ345854	PQ428462	PQ368664	PQ341124	PQ428509	PQ428556	PQ428603	PQ428650	PQ428697	PQ367521	PQ428744	PQ428791	PQ428838	PQ367568	PQ367615
	K22	PQ345873	PQ428481	PQ368665	PQ341143	PQ428528	PQ428575	PQ428622	PQ428669	PQ428716	PQ367540	PQ428763	PQ428810	PQ428857	PQ367587	PQ367634
	Hbb	PQ345868	PQ428476	PQ368668	PQ341138	PQ428523	PQ428570	PQ428617	PQ428664	PQ428711	PQ367535	PQ428758	PQ428805	PQ428852	PQ367582	PQ367629
*H. georgiana*	Kesha	PQ345874	PQ428482	PQ368666	PQ341144	PQ428529	PQ428576	PQ428623	PQ428670	PQ428717	PQ367541	PQ428764	PQ428811	PQ428858	PQ367588	PQ367635
	Point Reyes	PQ345886	PQ428494	PQ368667	PQ341156	PQ428541	PQ428588	PQ428635	PQ428682	PQ428729	PQ367553	PQ428776	PQ428823	PQ428870	PQ367600	PQ367647
	AB, HeM	PQ345855	PQ428463	PQ368669	PQ341125	PQ428510	PQ428557	PQ428604	PQ428651	PQ428698	PQ367522	PQ428745	PQ428792	PQ428839	PQ367569	PQ367616
*H. indica*	Bartow	PQ345857	PQ428465	PQ368670	PQ341127	PQ428512	PQ428559	PQ428606	PQ428653	PQ428700	PQ367524	PQ428747	PQ428794	PQ428841	PQ367571	PQ367618
	IARI, IARI-EPN-Hms1	PQ345871	PQ428479	PQ368671	PQ341141	PQ428526	PQ428573	PQ428620	PQ428667	PQ428714	PQ367538	PQ428761	PQ428808	PQ428855	PQ367585	PQ367632
	LN2	PQ345875	PQ428483	PQ368672	PQ341145	PQ428530	PQ428577	PQ428624	PQ428671	PQ428718	PQ367542	PQ428765	PQ428812	PQ428859	PQ367589	PQ367636
	Rebecca	PQ345890	PQ428498	PQ368673	PQ341160	PQ428545	PQ428592	PQ428639	PQ428686	PQ428733	PQ367557	PQ428780	PQ428827	PQ428874	PQ367604	PQ367651
*H. megidis*	UK211	PQ345897	PQ428505	PQ368674	PQ341167	PQ428552	PQ428599	PQ428646	PQ428693	PQ428740	PQ367564	PQ428787	PQ428834	PQ428881	PQ367611	PQ367658
*H. noenieputensis*	SF669	PQ345895	PQ428503	PQ368675	PQ341165	PQ428550	PQ428597	PQ428644	PQ428691	PQ428738	PQ367562	PQ428785	PQ428832	PQ428879	PQ367609	PQ367656
*H. ruandica*	Rw18_M-Hr1a, Ma	PQ345878	PQ428486	PQ368676	PQ341148	PQ428533	PQ428580	PQ428627	PQ428674	PQ428721	PQ367545	PQ428768	PQ428815	PQ428862	PQ367592	PQ367639
	Rw18_M-Hr1b, Mb	PQ345879	PQ428487	PQ368677	PQ341149	PQ428534	PQ428581	PQ428628	PQ428675	PQ428722	PQ367546	PQ428769	PQ428816	PQ428863	PQ367593	PQ367640
*H. safricana*	SF281	PQ345894	PQ428502	PQ368678	PQ341164	PQ428549	PQ428596	PQ428643	PQ428690	PQ428737	PQ367561	PQ428784	PQ428831	PQ428878	PQ367608	PQ367655
*H. taysearae*	Benin	PQ345858	PQ428466	PQ368685	PQ341128	PQ428513	PQ428560	PQ428607	PQ428654	PQ428701	PQ367525	PQ428748	PQ428795	PQ428842	PQ367572	PQ367619
*H. zacatecana*	MEX-39, MEX39	PQ345880	PQ428488	PQ368686	PQ341150	PQ428535	PQ428582	PQ428629	PQ428676	PQ428723	PQ367547	PQ428770	PQ428817	PQ428864	PQ367594	PQ367641
	MEX-41, MEX41	PQ345881	PQ428489	PQ368679	PQ341151	PQ428536	PQ428583	PQ428630	PQ428677	PQ428724	PQ367548	PQ428771	PQ428818	PQ428865	PQ367595	PQ367642
*H. zealandica*	Blue, SF41	PQ345859	PQ428467	PQ368681	PQ341129	PQ428514	PQ428561	PQ428608	PQ428655	PQ428702	PQ367526	PQ428749	PQ428796	PQ428843	PQ367573	PQ367620
	Green, MJ2C	PQ345865	PQ428473	PQ368680	PQ341135	PQ428520	PQ428567	PQ428614	PQ428661	PQ428708	PQ367532	PQ428755	PQ428802	PQ428849	PQ367579	PQ367626
*Heterorhabditis* sp.	CRI-LC, CRILC	PQ345863	PQ428471	PQ368682	PQ341133	PQ428518	PQ428565	PQ428612	PQ428659	PQ428706	PQ367530	PQ428753	PQ428800	PQ428847	PQ367577	PQ367624

**Table S4:** National Center for Biotechnology Information (NCBI) accession numbers of the *Photorhabdus* sequences used in this study. Sequences generated in this study are shown in bold.

**Strain**	**Genome**
*P. aballayi* APURE^T^	JAPFCD01
*P. africana* CRI-LC^T^	JAXBVE01
*P. aegyptia* BA1^T^	JFGV01
*P. akhurstii subsp. akhurstii* DSM 15138^T^	RCWE01
*P. akhurstii subsp. bharatensis* H3^T^	PUWU01
*P. antumapuensis* UCH-936^T^	JAHZMK01
*P. australis subsp. thailandensis* PB68.1^T^	LOMY01
*P. australis subsp. australis* DSM 17609^T^	JONO01
*P. asymbiotica* ATCC 43949^T^	RBLJ01
*P. bodei* LJ24-63^T^	NSCM01
*P. caribbeanensis* DSM 22391^T^	RCWB01
*P. cinerea* DSM 19724^T^	PUJW01
*P. hainanensis* DSM 22397^T^	RCWD01
*P. heterorhabditis subsp. aluminescens* Q614^T^	JABBCS01
*P. heterorhabditis subsp. heterorhabditis* SF41^T^	RCWA01
*P. hindustanensis* H1^T^	PUWT01
*P. kayaii* DSM 15194^T^	JAJAFZ01
*P. khanii subsp. khanii* DSM 3369^T^	AYSJ01
*P. khanii subsp. guanajuatensis* MEX20-17^T^	PUJY01
*P. kleinii* DSM 23513^T^	JAJAFY01
*P. laumondii subsp. clarkei* BOJ-47^T^	NSCI01
*P. laumondii subsp. laumondii* TT01^T^	WSFH01
*P. luminescens subsp. luminescens* ATCC 29999^T^	FMWJ01
*P. luminescens subsp. mexicana* MEX47-22^T^	PUJX01
*P. luminescens subsp. venezuelensis* JAR^T^	JAPFFZ01
*P. namnaonensis* PB45.5^T^	LOIC01
*P. noenieputensis* DSM 25462^T^	RCWC01
*P. stackebrandtii* DSM 23271^T^	PUJV01
*P. tasmaniensis* DSM 22387^T^	PUJU01
***P. tasmaniensis* UOH-32**	**XXXXX**
*P. temperata* DSM 14550^T^	JAJAFX01
*P. thracensis* DSM 15199^T^	CP011104
*P. viridis* Green^T^	JBEJZY01

**Table S6:** Pairwise comparison of digital DNA–DNA Hybridization (dDDH) scores (%) of *P. tasmaniensis* UOH-032 and all the *Photorhabdus* type strains with validly published names. Accession numbers of gene sequences used are shown in Table S4.

**Strain**	**dDDH (%)**
*P. aballayi sp. nov.* APURE^T^	30.7
*P. aegyptia* BA1^T^	31.5
*P. africana* CRI-LC^T^	32.3
*P. akhurstii subsp. akhurstii* DSM 15138^T^	31.2
*P. akhurstii subsp. bharatensis* H3^T^	31.3
*P. antumapuensis* UCH-936^T^	32.7
*P. asymbiotica* DSM 15149^T^	30.7
*P. australis subsp. australis* DSM 17609^T^	30.5
*P. australis subsp. thailandensis* PB68.1^T^	30.8
*P. bodei* LJ24-63^T^	32.6
*P. caribbeanensis* HG29^T^	30.8
*P. cinerea* DSM 19724^T^	31.7
*P. hainanensis* DSM 22397^T^	31.1
*P. heterorhabditis subsp. aluminescens* Q614^T^	32.7
*P. heterorhabditis subsp. heterorhabditis* SF41^T^	31.7
*P. hindustanensis* H1^T^	31.1
*P. kayaii* DSM 15194^T^	32.6
*P. khanii subsp. guanajuatensis* MEX20-17^T^	48.1
*P. khanii subsp. khanii* DSM 3369^T^	47.6
*P. kleinii* DSM 23513^T^	32
*P. laumondii subsp. clarkei* BOJ-47^T^	32.1
*P. laumondii subsp. laumondii* TT01^T^	31.9
*P. luminescens subsp. luminescens* ATCC 29999^T^	30.8
*P. luminescens subsp. mexicana* MEX47-22^T^	30.7
*P. luminescens subsp. venezuelensis* JAR^T^	30.7
*P. namnaonensis* PB45.5^T^	31.1
*P. noenieputensis* DSM 25462^T^	31.1
*P. stackebrandtii* DSM 23271^T^	47.1
***P. tasmaniensis* DSM 22387^T^**	**85.3**
*P. temperata* DSM 14550^T^	51.1
*P. thracensis* DSM 15199^T^	50.8
*P. viridis* Green^T^	49

## References

[j_jofnem-2025-0045_ref_001] Andaló V., Nguyen K. B., Moino A. (2006). *Heterorhabditis amazonensis* n. sp. (Rhabditida: Heterorhabditidae) from Amazonas, Brazil. Nematology: International Journal of Fundamental and Applied Nematological Research.

[j_jofnem-2025-0045_ref_002] Astashyn A., Tvedte E. S., Sweeney D., Sapojnikov V., Bouk N., Joukov V., Mozes E., Strope P. K., Sylla P. M., Wagner L., Bidwell S. L., Brown L. C., Clark K., Davis E. W., Smith-White B., Hlavina W., Pruitt K. D., Schneider V. A., Murphy T. D. (2024). Rapid and sensitive detection of genome contamination at scale with FCS-GX. Genome Biology.

[j_jofnem-2025-0045_ref_003] Auch A. F., Klenk H. P., Göker M. (2010a). Standard operating procedure for calculating genome-to-genome distances based on high-scoring segment pairs. Standards in Genomic Sciences.

[j_jofnem-2025-0045_ref_004] Auch A. F., Von Jan M., Klenk H. P., Göker M. (2010b). Digital DNA-DNA hybridization for microbial species delineation by means of genome-to-genome sequence comparison. Standards in Genomic Sciences.

[j_jofnem-2025-0045_ref_005] Bankevich A., Nurk S., Antipov D., Gurevich A. A., Dvorkin M., Kulikov A. S., Lesin V. M., Nikolenko S. I., Pham S., Prjibelski A. D., Pyshkin A. V., Sirotkin A. V., Vyahhi N., Tesler G., Alekseyev M. A., Pevzner P. A. (2012). SPAdes: A new genome assembly algorithm and its applications to single-cell sequencing. Journal of Computational Biology.

[j_jofnem-2025-0045_ref_006] Bedding R. A., Akhurst R. J. (1975). A simple technique for the detection of insect parasitic rhabditid nematodes in soil. Nematologica.

[j_jofnem-2025-0045_ref_007] Bernt M., Donath A., Jühling F., Externbrink F., Florentz C., Fritzsch G., Pütz J., Middendorf M., Stadler P. F. (2013). MITOS: Improved de novo metazoan mitochondrial genome annotation. Molecular Phylogenetics and Evolution.

[j_jofnem-2025-0045_ref_008] Bhat A. H., Machado R. A. R., Abolafia J., Ruiz-Cuenca A. N., Askary T. H., Ameen F., Dass W. M. (2023). Taxonomic and molecular characterization of a new entomopathogenic nematode species, *Heterorhabditis casmirica* n. sp., and whole genome sequencing of its associated bacterial symbiont. Parasites and Vectors.

[j_jofnem-2025-0045_ref_009] Bolger A. M., Lohse M., Usadel B. (2014). Trimmomatic: a flexible trimmer for Illumina sequence data. Bioinformatics.

[j_jofnem-2025-0045_ref_010] Cantarel B. L., Korf I., Robb S. M. C., Parra G., Ross E., Moore B., Holt C., Sánchez Alvarado A., Yandell M. (2008). MAKER: An easy-to-use annotation pipeline designed for emerging model organism genomes. Genome Research.

[j_jofnem-2025-0045_ref_011] Chen S., Zhou Y., Chen Y., Gu J. (2018). Fastp: An ultra-fast all-in-one FASTQ preprocessor. Bioinformatics (Oxford, England).

[j_jofnem-2025-0045_ref_012] Chevenet F., Brun C., Bañuls A. L., Jacq B., Christen R. (2006). TreeDyn: Towards dynamic graphics and annotations for analyses of trees. BMC Bioinformatics.

[j_jofnem-2025-0045_ref_013] Courtney W. D., Polley D., Miller V. L. (1955). TAF, an improved fixative in nematode technique. Plant Disease Reporter.

[j_jofnem-2025-0045_ref_014] Dhakal M., Nguyen K. B., Hunt D. J., Ehlers R. U., Spiridonov S. E., Subbotin S. A. (2020). Molecular identification, phylogeny and phylogeography of the entomopathogenic nematodes of the genus *Heterorhabditis* Poinar, 1976: A multigene approach. Nematology: International Journal of Fundamental and Applied Nematological Research.

[j_jofnem-2025-0045_ref_015] Dierckxsens N., Mardulyn P., Smits G. (2016). NOVOPlasty: *De novo* assembly of organelle genomes from whole genome data. Nucleic Acids Research.

[j_jofnem-2025-0045_ref_016] Edgar R. C. (2004). MUSCLE: Multiple sequence alignment with high accuracy and high throughput. Nucleic Acids Research.

[j_jofnem-2025-0045_ref_017] Edgington S., Buddie A. G., Moore D., France A., Merino L., Hunt D. J. (2011). *Heterorhabditis atacamensis* n. sp. (Nematoda: Heterorhabditidae), a new entomopathogenic nematode from the Atacama Desert, Chile. Journal of Helminthology.

[j_jofnem-2025-0045_ref_018] Emms D. M., Kelly S. (2019). OrthoFinder: Phylogenetic orthology inference for comparative genomics. Genome Biology.

[j_jofnem-2025-0045_ref_019] Griffin C. T., Downes M. J., Block W. (1990). Tests of Antarctic soils for insect parasitic nematodes. Antarctic Science.

[j_jofnem-2025-0045_ref_020] Katoh K., Kuma K., Toh H., Miyata T. (2005). MAFFT version 5: Improvement in accuracy of multiple sequence alignment. Nucleic Acids Research.

[j_jofnem-2025-0045_ref_021] Kimura M. (1980). A simple method for estimating evolutionary rates of base substitutions through comparative studies of nucleotide sequences. Journal of Molecular Evolution.

[j_jofnem-2025-0045_ref_022] Kumar S., Stecher G., Tamura K. (2016). MEGA7: Molecular Evolutionary Genetics Analysis version 7.0 for bigger datasets. Molecular Biology and Evolution.

[j_jofnem-2025-0045_ref_023] Langmead B., Salzberg S. L. (2012). Fast gapped-read alignment with Bowtie 2. Nature Methods.

[j_jofnem-2025-0045_ref_024] Lankin G., Santiagos A., Hermosilla M., Aballay E., San-Blas E. (2022). A novel approach for the biological control of invasive *Bagrada* bugs with entomopathogenic nematodes. Journal of Pest Science.

[j_jofnem-2025-0045_ref_025] Letunic I., Bork P. (2016). Interactive tree of life (iTOL) v3: An online tool for the display and annotation of phylogenetic and other trees. Nucleic Acids Research.

[j_jofnem-2025-0045_ref_026] Machado R. A. R., Abolafia J., Robles M. C., Ruiz-Cuenca A. N., Bhat A. H., Shokoohi E., Půža V., Zhang X., Erb M., Robert C. A. M., Hibbard B. (2025a). Description of *Heterorhabditis americana* n. sp. (Rhabditida, Heterorhabditidae), a new entomopathogenic nematode species isolated in North America. Parasites and Vectors.

[j_jofnem-2025-0045_ref_027] Machado R. A. R., Bhat A. H., Abolafia J., Muller A., Bruno P., Fallet P., Arce C. C. M., Turlings T. C. J., Bernal J. S., Kajuga J., Waweru B., Toepfer S. (2021). Multi-locus phylogenetic analyses uncover species boundaries and reveal the occurrence of two new entomopathogenic nematode species, *Heterorhabditis ruandica* n. sp. and *Heterorhabditis zacatecana* n. sp.. Journal of Nematology.

[j_jofnem-2025-0045_ref_028] Machado R. A. R., Muller A., Hiltmann A., Bhat A. H., Půža V., Malan A. P., Castaneda-Alvarez C., San-Blas E., Duncan L. W., Shapiro-Ilan D., Karimi J., Lalramliana, Lalramnghaki H. C., Baimey H. (2025b). Genome-wide analyses provide insights into genetic variation, phylo- and co-phylogenetic relationships, and biogeography of the entomopathogenic nematode genus *Heterorhabditis*. Molecular Phylogenetics and Evolution.

[j_jofnem-2025-0045_ref_029] Machado R. A. R., Wüthrich D., Kuhnert P., Arce C. C. M., Thönen L., Ruiz C., Zhang X., Robert C. A. M., Karimi J., Kamali S., Ma J., Bruggmann R., Erb M. (2018). Whole-genome-based revisit of *Photorhabdus* phylogeny: Proposal for the elevation of most *Photorhabdus* subspecies to the species level and description of one novel species *Photorhabdus bodei* sp. nov., and one novel subspecies *Photorhabdus laumondii* subsp. clarkei subsp. nov.. International Journal of Systematic and Evolutionary Microbiology.

[j_jofnem-2025-0045_ref_030] Meier-Kolthoff J. P., Auch A. F., Klenk H. P., Göker M. (2013). Genome sequence-based species delimitation with confidence intervals and improved distance functions. BMC Bioinformatics.

[j_jofnem-2025-0045_ref_031] Meier-Kolthoff J. P., Hahnke R. L., Petersen J., Scheuner C., Michael V., Fiebig A., Rohde C., Rohde M., Fartmann B., Goodwin L. A., Chertkov O., Reddy T., Pati A., Ivanova N. N., Markowitz V., Kyrpides N. C., Woyke T., Göker M., Klenk H. P. (2014). Complete genome sequence of DSM 30083T, the type strain (U5/41T) of *Escherichia coli*, and a proposal for delineating subspecies in microbial taxonomy. Standards in Genomic Sciences.

[j_jofnem-2025-0045_ref_032] Nguyen K. B., Nguyen K.B., Hunt D.J. (2007). Methodology, morphology and identification. Entomopathogenic nematodes: systematics, phylogeny and bacterial symbionts. Nematology Monographs and Perspectives.

[j_jofnem-2025-0045_ref_033] Nguyen K. B., Shapiro-Ilan D., Mbata G. N. (2008). *Heterorhabditis georgiana* n. sp. (Rhabditida: Heterorhabditidae) from Georgia, USA. Nematology: International Journal of Fundamental and Applied Nematological Research.

[j_jofnem-2025-0045_ref_034] Parks D. H., Imelfort M., Skennerton C. T., Hugenholtz P., Tyson G. W. (2015). CheckM: Assessing the quality of microbial genomes recovered from isolates, single cells, and metagenomes. Genome Research.

[j_jofnem-2025-0045_ref_035] Pereira C. (1937). *Rhabditis hambletoni* n. sp., nema apparentemente semiparasito da Broca do algodoeiro (*Gasterocercodes brasiliensis*). Archivos Instituto Biologico.

[j_jofnem-2025-0045_ref_036] Poinar G. O. (1975). Description and biology of a new insect parasitic rhabditoid, *Heterorhabditis bacteriophora* n. gen., n. sp. (Rhabditida; Heterorhabditidae n. fam.). Nematologica.

[j_jofnem-2025-0045_ref_037] Poinar G. O., Karunakar G. K., David H. (1992). *Heterorhabditis indicus* n. sp. (Rhabditida: nematoda) from India: Separation of *Heterorhabditis* spp. by infective juveniles. Fundamental and Applied Nematology.

[j_jofnem-2025-0045_ref_038] Price M. N., Dehal P. S., Arkin A. P. (2010). FastTree 2 – approximately maximum-likelihood trees for large alignments. Plos One.

[j_jofnem-2025-0045_ref_039] Půža V., Machado R. A. R. (2024). Systematics and phylogeny of the entomopathogenic nematobacterial complexes *Steinernema–Xenorhabdus* and *Heterorhabditis–Photorhabdus*. Zoological Letters.

[j_jofnem-2025-0045_ref_040] Půža V., Machado R. A. R., Malan A. P. (2025). Systematics, diversity and biogeography of entomopathogenic nematodes and their bacterial symbionts. Journal of Invertebrate Pathology.

[j_jofnem-2025-0045_ref_041] San-Blas E., Campos-Herrera R., Dolinski C., Monteiro C., Andaló V., Leite L. G., Rodríguez M. G., Morales-Montero P., Sáenz-Aponte A., Cedano C., López-Nuñez J. C., Del Valle E., Doucet M., Lax P., Navarro P. D., Báez F., Llumiquinga P., Ruiz-Vega J., Guerra-Moreno A., Stock S. P. (2019). Entomopathogenic nematology in Latin America: A brief history, current research and future prospects. Journal of Invertebrate Pathology.

[j_jofnem-2025-0045_ref_042] Seppey M., Manni M., Zdobnov E. M., Kollmar M. (2019). BUSCO: Assessing genome assembly and annotation completeness. Gene prediction.

[j_jofnem-2025-0045_ref_043] van Lenteren J. C., Bueno V. H. P., Betiol W. (2025). Latin America has the largest area under augmentative biological control worldwide, mainly with applications in open field crops. Biological Control.

[j_jofnem-2025-0045_ref_044] Walker B. J., Abeel T., Shea T., Priest M., Abouelliel A., Sakthikumar S., Cuomo C. A., Zeng Q., Wortman J., Young S. K., Earl A. M. (2014). Pilon: An integrated tool for comprehensive microbial variant detection and genome assembly improvement. Plos One.

[j_jofnem-2025-0045_ref_045] Wayne L. G., Brenner D. J., Colwell R. R., Grimont P. A. D., Kandler O., Krichevsky M. I., Moore L. H., Moore W. E. C., Murray R. G. E., Stackebrandt E., Starr M. P., Truper H. G. (1987). Report of the ad hoc committee on reconciliation of approaches to bacterial systematics. International Journal of Systematic Bacteriology.

[j_jofnem-2025-0045_ref_046] White G. F. (1927). A method for obtaining infective nematode larvae from cultures. Science.

[j_jofnem-2025-0045_ref_047] Wick R. R., Judd L. M., Gorrie C. L., Holt K. E. (2017). Unicycler: Resolving bacterial genome assemblies from short and long sequencing reads. PLoS Computational Biology.

